# Effects of aspirin and non-aspirin nonsteroidal anti-inflammatory drugs on the incidence of recurrent colorectal adenomas: a systematic review with meta-analysis and trial sequential analysis of randomized clinical trials

**DOI:** 10.1186/s12885-017-3757-8

**Published:** 2017-11-14

**Authors:** Sajesh K. Veettil, Kean Ghee Lim, Siew Mooi Ching, Surasak Saokaew, Pochamana Phisalprapa, Nathorn Chaiyakunapruk

**Affiliations:** 1 School of Pharmacy/School of Postgraduate Studies, International Medical University, Kuala Lumpur, Malaysia; 2 Clinical School, Department of Surgery, International Medical University, Seremban, Negeri Sembilan Malaysia; 3 Department of Family Medicine, Faculty of Medicine and Health Sciences, Serdang, Malaysia; 4 Malaysian Research Institute on Ageing, Universiti Putra Malaysia, Serdang, Malaysia; 5 Center of Health Outcomes Research and Therapeutic Safety (Cohorts), School of Pharmaceutical Sciences, University of Phayao, Phayao, Thailand; 6 School of Pharmacy, Monash University Malaysia, Jalan Lagoon Selatan, 46150 Bandar Sunway, Selangor Malaysia; 7 Center of Pharmaceutical Outcomes Research, Department of Pharmacy Practice, Faculty of Pharmaceutical Sciences, Naresuan University, Naresuan University, Phitsanulok, Thailand; 8 Unit of Excellence on Herbal Medicine, School of Pharmaceutical Sciences, University of Phayao, Phayao, Thailand; 9 Division of Ambulatory Medicine, Department of Medicine, Faculty of Medicine Siriraj Hospital, Mahidol University, Bangkok, Thailand; 10 School of Pharmacy, University of Wisconsin, Madison, USA; 11 Asian Centre for Evidence Synthesis in Population, Implementation and Clinical Outcomes (PICO), Health and Well-being Cluster, Global Asia in the 21st Century (GA21) Platform, Monash University Malaysia, Bandar Sunway, Selangor Malaysia

**Keywords:** Colorectal adenomas, Aspirin, Anti-inflammatory agents, Non-steroidal, Systematic review, Meta-analysis, Randomized controlled trials, Trial sequential analysis

## Abstract

**Background:**

Beneficial effects of aspirin and non-aspirin nonsteroidal anti-inflammatory drugs (NSAIDs) against recurrent colorectal adenomas have been documented in systematic reviews; however, the results have not been conclusive. Uncertainty remains about the appropriate dose of aspirin for adenoma prevention. The persistence of the protective effect of NSAIDs against recurrent adenomas after treatment cessation is yet to be established.

**Methods:**

Our objective was to update and systematically evaluate the evidence for aspirin and other NSAIDs on the incidence of recurrent colorectal adenomas taking into consideration the risks of random error and to appraise the quality of evidence using GRADE (The Grading of Recommendations, Assessment, Development and Evaluation) approach. Retrieved trials were evaluated using Cochrane risk of bias instrument. Meta-analytic estimates were calculated with random-effects model and random errors were evaluated with trial sequential analysis (TSA).

**Results:**

In patients with a previous history of colorectal cancer or adenomas, low-dose aspirin (80–160 mg/day) compared to placebo taken for 2 to 4 years reduces the risk of recurrent colorectal adenomas (relative risk (RR), 0.80 [95% CI (confidence interval), 0.70–0.92]). TSA indicated a firm evidence for this beneficial effect. The evidence indicated moderate GRADE quality. Low-dose aspirin also reduces the recurrence of advanced adenomas (RR, 0.66 [95% CI, 0.44–0.99]); however, TSA indicated lack of firm evidence for a beneficial effect. High-dose aspirin (300–325 mg/day) did not statistically reduce the recurrent adenomas (RR, 0.90 [95% CI, 0.68–1.18]). Cyclooxygenase-2 (COX-2) inhibitors (e.g. celecoxib 400 mg/day) were associated with a significant decrease in the recurrence of both adenomas (RR, 0.66 [95% CI, 0.59–0.72]) and advanced adenomas (RR, 0.45 [95% CI, 0.33–0.57]); however, this association did not persist and there was a trend of an increased risk of recurrent adenomas observed 2 years after the withdrawal.

**Conclusion:**

Our findings confirm the beneficial effect of low-dose aspirin on recurrence of any adenomas; however, effect on advanced adenomas was inconclusive. COX-2 inhibitors seem to be more effective in preventing recurrence of adenomas; however, there was a trend of an increased risk of recurrence of adenomas observed after discontinuing regular use.

**Electronic supplementary material:**

The online version of this article (10.1186/s12885-017-3757-8) contains supplementary material, which is available to authorized users.

## Background

Colorectal adenomas are prominent precursor lesions of the colorectal cancer [1]. Majority of colorectal cancers develop from adenomas, through a series of genetic changes (adenoma-carcinoma sequence) during a time interval of at least 5–10 years [1]. When adenomas are large or villous or severely dysplastic (defined as advanced adenomas), the risk of subsequent cancer is highest [1]. Adenomas are considered a reasonable surrogate end point for trials in this area particularly among those with a past history of colorectal cancer or adenomas where rates of recurrence are known to be higher than the general population [2, 3]. Favourable effect of aspirin and other nonsteroidal anti-inflammatory drugs (NSAIDs), including cyclooxygenase- 2 (COX-2) inhibitors, on recurrent colorectal adenomas have been reported in many observational studies and randomized controlled trials (RCTs) [4–6].

Published systematic reviews [5–7] and meta-analyses [8–11] based on the results from RCTs [12–17] propose that aspirin at any doses decreases the risk of recurrent colorectal adenomas. On the other hand, use of aspirin was associated with a dose-related increase in occurrence of gastrointestinal complications [5]. Low-dose aspirin used for cardiovascular protection may provide an additional advantage as the balance of benefits and risks seems to be more favourable [5, 18, 19]. Previous two meta-analyses [8, 9], demonstrated a moderate beneficial effect of low-dose aspirin on preventing recurrent adenomas. However, the authors did not find statistically significant evidence to support a protective role of low-dose aspirin on recurrent advanced adenomas. More recently, additional studies [16, 17] have been published (the latest report of APACC trial (2012) and the Ishikawa (2014) trial) necessitates an update of the previous systematic reviews to re-examine the evidence. Moreover, previous meta-analyses [8–11] did not reflect the risks of random errors, and did not grade the quality of evidence using GRADE (The Grading of Recommendations, Assessment, Development and Evaluation) approach for reliability [20, 21]. When a meta-analysis comprises a small number of RCTs and patients, random errors can lead to a deceptive conclusions [21, 22]. Some ‘positive’ meta-analytic results may be due to the play of chance (random error) rather than due to some underlying ‘true’ intervention effect [21, 22]. Trial sequential analysis (TSA) considers the risks of random errors and demonstrate the required sample size and boundaries that consider whether the evidence in a meta-analysis is conclusive [21]. This emphasizes the importance of updating the summary of effects of aspirin in different doses on the incidence of recurrent adenomas and advanced adenomas using recently published trials and taking into account the risks of random errors.

Moreover, some observational studies suggest that the protective effect of NSAIDs against recurrent adenomas may disappear after discontinuing regular use [4, 23], and the data regarding the tenacity of the effect are not extensive [24, 25]. Recent post-trial follow-up results from Pre SAP study [26] and APC trial [27] reported the absence of a protective effect of COX-2 inhibitors on the incidence of recurrent adenomas after drug withdrawal. Moreover, a statistically significant increased risk of adenoma was reported in the post-trial follow-up of the rofecoxib trial after 1 year treatment cessation [28]. These results emphasize the importance of investigating effects of NSAIDs on the incidence of recurrent adenomas during treatment and after withdrawal.

The objective of this review was to systematically update the effects of aspirin at different doses and non-aspirin NSAIDs on recurrent colorectal adenoma prevention. To quantify the reliable and conclusive evidence of aspirin, we performed meta-analyses coupled with trial sequential analyses. We also summarized the evidence using the GRADE approach. Lastly, we examined the effect of aspirin/non-aspirin NSAIDs on the risk of recurrent adenomas after the removal of the drug.

## Methods

### Design and data sources

This study was conducted as a part of a systematic review and network meta-analysis of chemopreventive interventions for colorectal cancer which has been registered (registration number: CRD42015025849) with the PROSPERO (International Prospective Register of Systematic Reviews), previously [29]. A complete description of the parent study design and methods has been published elsewhere [30]. We used the Cochrane Handbook for Systematic Reviews of Interventions for the preparation and conduct of this meta-analysis [31]. The writing adhered strictly to the Preferred Reporting Items for Systematic reviews and Meta-Analyses (PRISMA) guidelines [32].

We identified relevant studies by a systematic search of MEDLINE 2008 to September 2016 (Via Ovid), MEDLINE In-Process & Other Non-Indexed Citations (Via Ovid), Embase 2008 to September 2016 (Via Ovid), Cochrane CENTRAL Register of Controlled Trials (September 2016, Via Ovid), CINAHL plus (January 2008 to September 2016), International Pharmaceutical Abstracts (September 2016) and clinicaltrials.gov website (September 2016). We developed the search strategy in MEDLINE and modified it for other databases (Additional file 1: Table S1, published online). The search was restricted to studies published from 2008 onwards because studies published up to 2007 could be identified from the published systematic reviews [4–10]. We manually checked the reference lists of published systematic reviews and identified articles to categorise the studies which were not captured by existing database searches.

Studies included were RCTs and post-trial reports with a follow-up at least 1 year and met the following criteria: participants were adults with history of colorectal cancer or adenomas; interventions were aspirin or non-aspirin NSAIDs at any dose; comparators were placebo or no treatment; and primary outcomes were the incidences of any recurrent colorectal adenomas and of advanced adenomas. We excluded RCTs that reported the efficacy of combination of aspirin or non-aspirin NSAIDs with other chemopreventive agents with evidence of efficacy against recurrent colorectal adenomas and trials in adults with history of familial cancer syndromes (such as Lynch syndrome).

### Data extraction and quality assessment

Requisite data were extracted independently and in duplicate by two reviewers into a data extraction form (SKV, SMC). Two reviewers (SKV, KGL) independently assessed the risk of bias within each study by using a Cochrane risk of bias instrument [31, 33]. We evaluated sequence generation, allocation concealment, blinding of participants and personnel, blinding of outcome assessment, incomplete outcome data, selective outcome reporting, and other sources of bias. Reviewers resolved disagreements by discussion, and one of two arbitrators adjudicated any unsolved disagreements. When risks of bias vary across included studies, we will restrict analyses to studies at low risk of bias with justification for reporting the best evidence [31, 33].

### Statistical analysis

Quantitative synthesis was conducted by using random-effects model or inverse-variance weighting. Results were combined numerically only if clinically and statistically appropriate. In such cases, a narrative overview of the findings of included studies was presented with tabular summaries of extracted data. Heterogeneity between trials was assessed by considering the I^2^ statistic. An I^2^ estimate greater than or equal to 50% was interpreted as evidence of a substantial levels of heterogeneity [31]. Analyses were performed using STATA 14.1 software. We assessed publication bias using funnel plot asymmetry testing and Egger’s regression test [34].

Meta-analyses might result in type-I errors owing to an increased risk of random error when only few RCTs and less number of patients are involved, and due to continuous significance testing when a cumulative meta-analysis is updated with new RCTs [21, 22]. Therefore, to assesses the risks of random errors, we performed trial sequential analysis (TSA) using TSA software package (available at http://www.ctu.dk) [35], which combines information size estimation for meta-analysis (cumulated sample size of included trials) with an adjusted threshold for statistical significance in the cumulative meta-analysis. Trial sequential analysis provides the necessary sample size for our meta-analysis and boundaries that determine whether the evidence in our meta-analysis is reliable and conclusive [21]. Where the study did not report the actual event data, or if we observed a meta-analysis with substantial levels of heterogeneity, we avoided performing trial sequential analysis.

The Grading of Recommendations, Assessment, Development and Evaluation (GRADE) approach was used to rate the quality of evidence of estimates (high, moderate, low, and very low) derived from meta-analyses using GRADEpro GDT software. Reviewers independently assessed the confidence in effect estimates for all outcomes using the following categories: risk of bias, inconsistency, indirectness, imprecision and publication bias [20, 36] (See Additional file 1: Table S2, published online).

## Results

### Study selection

Study selection, inclusion, and exclusion at each screening phase for the efficacy end points are described in Additional file 1: Figure S1 (a flow of study selection-published online). Five RCTs [12, 14–17] comparing aspirin versus placebo and three [28, 37, 38] for NSAIDs other than aspirin versus placebo for the prevention of recurrent colorectal adenomas in subjects with a previous history of colorectal cancer or adenomas met the eligibility criteria. Tables 1 and 2 describe the characteristics of included studies. Another three RCTs [13, 39, 40] were identified for aspirin and two [41, 42] for non-aspirin NSAID, but did not meet the eligibility criteria, and were excluded with reasons (See Additional file 1: Table S3, published online).

**Table 1:** Characteristics of RCTs and summary of effects of aspirin on the incidence of recurrent colorectal adenomas

Study, year, (study name)	Location	Duration of treatment (follow-up schedule)	Population	Interventions (Number of patients randomized, n)	Outcomes	% of randomized participants excluded from main analyses	Compliance to treatments. Mean percentage of study pills taken % (SD)	Summary of results
**Baron 2003** (Aspirin/Folate Polyp Prevention Study (AFPPS)) [12]	United States	≈3 years (3 years after the baseline examination)	Age - range, 21–81 years; % male: 64; subjects with history of adenomas; and documented clean colon post-polypectomy	Aspirin 81 mg/day (*n* = 169); aspirin 325 mg/day (*n* = 167); aspirin 81 mg/day and folic acid 1 mg/day (*n* = 175); aspirin 325 mg/day and folic acid 1 mg/day (*n* = 171); folic acid 1 mg/day (*n* = 170); placebo (n = 169).	**Primary outcome:** recurrent colorectal adenomas **Secondary outcomes:** numbers of colorectal adenomas and advanced adenomas (defined as those with tubule-villous adenomas (25 to 75% villous features), villous adenomas (more than 75% villous), large adenomas (at least 1 cm in diameter), severe dysplasia, or invasive cancer)	3% excluded from analysis as no follow-up colonoscopy	Aspirin any dose- 91.7 (18.8); aspirin 81 mg- 91.9 (18.8); aspirin 325 mg- 91.6 (18.7); placebo- 90.3 (20.5)	**Unadjusted relative Risk (95% CI):** **Aspirin any dose versus placebo** Any adenoma: 0.88 (0.77–1.02) Advanced adenoma: 0.71 (0.50–1.00) **Aspirin 81 mg versus placebo** Any adenoma: 0.81 (0.69–0.96) Advanced adenoma: 0.59 (0.38–0.92) **Aspirin 325 mg versus placebo** Any adenoma: 0.96 (0.81–1.13) Advanced adenoma: 0.83 (0.55–1.23)
**Sandler 2003** (The colorectal adenoma preventions study [Cancer and Leukemia Group B (CALGB) 9270] [15]	United States	≈3 years (Participants with early-stage disease at 4 years and all other participants at 3 years after the baseline examination)	Ages – range, 30–80 years; % male: 52; subjects with of histologically documented colon or rectal cancer with a low risk of recurrent disease; and documented clean colon post-polypectomy	Aspirin 325 mg/day (*n* = 317); placebo (*n* = 318)	**Primary outcome:** incidence of adenoma. **Secondary outcomes:** the size of the largest adenoma; the time to the detection of a first adenoma, and the proportion of patients with advanced adenomas (defined as those that were at least 1 cm in diameter or had villous components)	19% excluded from analysis as no follow-up colonoscopy	Aspirin- 79.4 (26.8); placebo- 74.9 (28.5)	**Adjusted relative risk (95% CI)** **Aspirin 325 mg versus placebo** Any adenoma: 0.65 (0.46–0.91) Advanced adenoma: Not reported in the study. Risk ratio reported by Cole et al. [8] -0.77 (0.29–2.05)
**Logan 2008,** (United Kingdom Colorectal Adenoma Prevention (ukCAP) trial) [14]	United Kingdom and Denmark	3 years (3 years after the baseline examination)	Age – mean,58 years; range, 28–75 years; % male: 56; subjects with history of colorectal adenoma 0.5 cm or greater; and documented clean colon post-polypectomy	Aspirin 300 mg/day (*n* = 236); folic acid 0.5 mg/day (*n* = 234); aspirin 300 mg/day and folic acid 0.5 mg/day (n = 236); placebo (*n* = 233)	**Primary outcome:** recurrent colorectal adenomas **Secondary outcomes:** number of adenomas detected and incidence of advanced colorectal neoplasia (advanced colorectal neoplasia defined as adenomas that were either 1 cm or larger in diameter, villous or tubule-villous, or showed severe dysplasia or invasive cancer)	10% excluded from analysis as no follow-up colonoscopy	Aspirin- 77.1 (35.2); placebo- 80.9 (31.6)	**Relative Risk (95% CI) - Aspirin 300 mg versus placebo** Any adenoma: 0.79 (0.63–0.99) Advanced adenoma: 0.63 (0.43–0.91)
**Benamouzig 2012** (Association pour la Prevention par l’ Aspirine du Cancer Colorectal (APACC) Study-4 year results) [17]	France	4 years (4 years after the baseline examination)	Age – range, 18–75 years; % male: 70; subjects with history of at least 3 adenomas irrespective of size, or at least one measuring 6 mm in diameter or more; and documented clean colon post-polypectomy	Aspirin 160 mg/day (*n* = 73); aspirin 300 mg/day (*n* = 67); placebo (*n* = 132) (Aspirin ≈ lysine acetylsalicylate)	**Primary outcomes:** recurrent colorectal adenomas, and the adenomatous polyp burden **Secondary outcomes:** mean numbers of recurrent adenomas and numbers of recurrent advanced adenomas (defined as those with a maximum diameter of at least 10 mm, at least 25% villous elements or evidence of high-grade dysplasia)	32% excluded from analysis as no follow-up colonoscopy at year 4	Aspirin – 88 (26); placebo- 88 (26)	**Aspirin any dose versus placebo** *(Relative risk, not reported)* Any adenoma: Aspirin at any dose-42/102 (41%); Aspirin 160 mg-15/55 (27%); Aspirin 300 mg – 27/47 (57%); Placebo-33/83 (40%); non-significant. Advanced adenoma: Aspirin at any dose-10/182 (10%); Aspirin 160 mg-6/55 (11%); Aspirin 300 mg – 4/47 (8.5%); Placebo-7/83 (7%); non-significant.
**Ishikawa 2014** [16]	Japan	2 years (2 years after the baseline examination)	Age - range, 40–70 years; % male: 79; subjects with history of colorectal adenomas and/or adenocarcinomas with invasions confined to the mucosa; and documented clean colon post-polypectomy	Aspirin (enteric-coated) 100 mg/day (*n* = 191); placebo (*n* = 198).	**Primary outcome:** incidence of adenoma or adenocarcinoma recurrence (advanced adenomas defined as high-grade dysplasias) **Secondary outcomes:** recurring tumor number, size and histology as well as the effects of lifestyle, such as smoking and alcohol drinking, and the frequency of adverse effects		not available (Stated “no significant difference between the two groups in compliance rates”)	**Adjusted odds ratio (OR)** **Aspirin 100 mg versus placebo** Any adenoma (reported as colorectal tumour): 0.60 (95% CI 0.36 to 0.98) Advanced adenoma: Adjusted OR not reported. [Reported incidence of high grade dysplasia: Aspirin-1/152 (0.7%); Placebo-2/159 (1.3%)]

**Table 2:** Characteristics of RCTs and summary of effects of non-aspirin NSAIDs on the incidence of recurrent colorectal adenomas

Study, year, (study name)	Location	Duration of treatment (follow-up schedule)	Population	Interventions (Number of patients randomized, n)	Outcomes	% of randomized participants excluded from main analyses	Compliance to treatments	Reported Results
Arber 2006 (The Prevention of Colorectal Sporadic Adenomatous Polyps (Pre SAP) study) [37]	Multi-national	≈3 years (1 and 3 years after the baseline examination)	Age – median, 61 years; range, 30–92 years; % male: 66; subjects with history of adenomas; and documented clean colon post-polypectomy.	Celecoxib 400 mg/day (*n* = 933); placebo (*n* = 628)	**Primary outcome:** adenoma recurrence at year 1, 3, or both **Secondary outcomes:** advanced adenomas recurrence at year 1, 3, or both; cardiovascular outcomes and adverse events (advanced adenoma defined as adenoma ≥1.0 cm (villous or tubule-villous histology); high-grade dysplasia; Intra-mucosal carcinoma or invasive cancer)	11% excluded from analysis as no follow-up colonoscopy at year 1 or year 3	78% of participants reported taking the majority of their study medications, with similar compliance between arms	**Relative Risk (95% CI)** **Celecoxib 400 mg versus placebo** Any adenoma: 0.64 (0.56–0.75) Advanced adenoma: 0.49 (0.33–0.73)
Bertagnolli 2006 (The Adenoma Prevention with Celecoxib (APC) trial) [38]	Multi-national	3 years (1 and 3 years after the baseline examination)	Age – median, 59 years; range, 31–88 years; % male: 68; subjects with history of adenomas; and documented clean colon post-polypectomy.	Celecoxib 400 mg/day (*n* = 685); Celecoxib 800 mg/day (*n* = 671); Placebo (*n* = 679)	**Primary outcome:** adenoma recurrence at year 1, 3, or both **Secondary outcomes:** advanced adenomas recurrence at year 1, 3, or both; number of adenomas; size of largest adenoma; adenoma burden; cardiovascular outcomes and adverse events (advanced adenoma defined as adenoma ≥1.0 cm (villous or tubule-villous histology); high-grade dysplasia; Intra-mucosal carcinoma or invasive cancer)	10% excluded from analysis as no follow-up colonoscopy at year 1 or year 3	≈ 68% of participants reported taking the majority of their study medications at least 80% of the time, with similar compliance between arms.	**Relative Risk (95% CI)** **Celecoxib 400 versus placebo** Any adenoma: 0.67 (0.59–0.77) Advanced adenoma: 0.43 (0.31–0.61) **Celecoxib 800 versus placebo** Any adenoma: 0.55 (0.48–0.64) Advanced adenoma: 0.34 (0.24–0.50)
								**Summary for celecoxib, 400 mg/day versus placebo** **Relative Risk (95% CI)** Any adenoma: 0.66 (0.59–0.72) Advanced adenoma: 0.45 (0.33–0.57)
Baron 2006 (The Adenomatous Polyp PRevention On Vioxx (APPROVe) Trial [28]	Multi-national	3 years (1 and 3 years after the baseline examination)	Age – mean, 59.4 years; range, 40–86 years; % male: 62.3%; subjects with history of adenomas; and documented clean colon post-polypectomy.	Rofecoxib 25 mg/day (*n* = 1277); placebo(*n* = 1293)	**Primary outcome:** ≥1 adenoma at year 1 or 3 on colonoscopy **Secondary outcomes:** number of adenomas; advanced adenomas; death; cardiovascular and gastrointestinal events (advanced adenoma defined as adenoma ≥1.0 cm (villous or tubule-villous histology); high-grade dysplasia; Intra-mucosal carcinoma or invasive cancer)	7% excluded from analysis as no follow-up colonoscopy	More than 87% of subjects reported taking at least 90% of their tablets in the third year of the trial	**Relative Risk (95% CI)** **Rofecoxib versus placebo** Any adenoma: 0.76 (0.69–0.83) Advanced adenoma: 0.56 (0.42–0.75)

Five post-trial studies [25–28, 43] were available to investigate the effect of drugs withdrawal on incidence of recurrent adenomas. Additional file 1: Table S4 describes the identified studies.

### Effect of aspirin on incidence of recurrent colorectal adenomas

Characteristics of the included studies and study participants are described in Table 1. Using the Cochrane risk of bias assessment tool, all five RCTs [12, 14–17] included in the meta-analysis had low risks of bias in most criteria (See Additional file 1: Table S5). The risk of bias graph and summary are illustrated in Additional file 1: Figure S2 (published online). Among the four studies [12, 14, 15, 17], compliance with the study treatments was generally good with a mean pill-taking compliance ranged from approximately 69% to approximately 92%; however, the study by Ishikawa et al., did not report compliance data (Table 1).

**Fig. 1:**
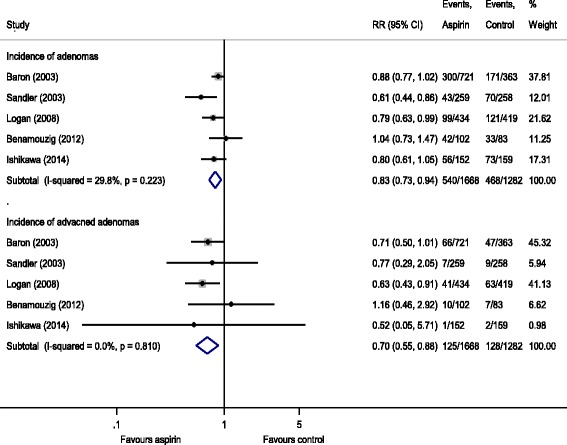
Incidence of recurrent adenomas and advanced adenomas in subjects with a history of colorectal cancer or adenomas randomized to aspirin (at any dose) vs. placebo/no intervention

Figure 1 summarizes the random-effects meta-analysis comparing aspirin in any dose (80 mg to 325 mg) to placebo. Among 2950 participants for whom follow-up colonoscopy results were available, adenomas were found in 540 (32%) of the 1668 participants allocated to any dose of aspirin and in 468 (37%) of the 1282 participants allocated to placebo. Quantitative pooling of results from these RCTs indicated that the use of aspirin in any dose lasting 2 to 4 years showed a statistically significant 17% relative risk reduction (RRR) in the recurrent risk of any adenomas (RR, 0.83 [95% CI 0.73 to 0.94]), with a moderate level of statistical heterogeneity (I^2^ = 29.8%). Among participants with a similar colonoscopic follow-up, advanced adenomas (defined in Table 1) were found in 125 (7.5%) participants allocated to any dose of aspirin and in 128 (10%) participants in the placebo group, which corresponded to a statistically significant RRR of 30% for aspirin in any dose (RR, 0.70 [95% CI 0.55 to 0.88]), with no heterogeneity (I^2^ = 0%).

### Subgroup analysis based on dose

**Fig. 2:**
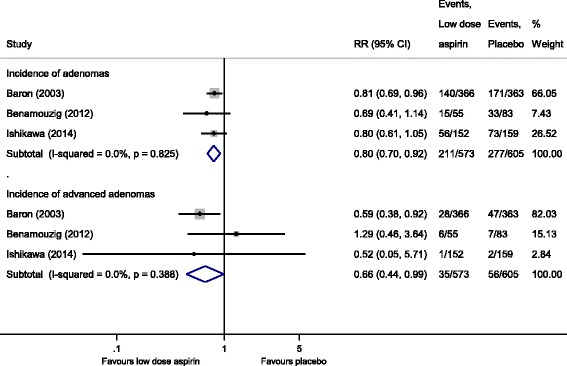
Incidence of recurrent adenomas and advanced adenomas in subjects with a history of colorectal cancer or adenomas randomized to low-dose aspirin vs. placebo/no intervention

**Fig. 3:**
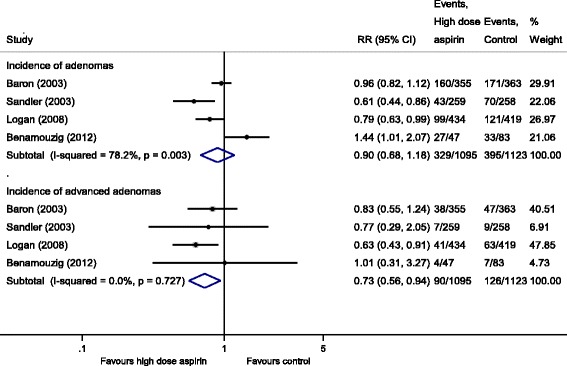
Incidence of recurrent adenomas and advanced adenomas in subjects with a history of colorectal cancer or adenomas randomized to high-dose aspirin vs. placebo/no intervention

When we stratified studies based on the dose of aspirin, pooling the three RCTs [12, 16, 17] showed that low-dose aspirin (80 to 160 mg/day), produced a statistically significant RRR of 20% for recurrence of any adenomas (RR, 0.80 [95% CI 0.70 to 0.92]) and 34% for advanced adenomas (RR, 0.66 [95% CI 0.44 to 0.99]), with no heterogeneity (I^2^ = 0%) (Fig. 2). Information regarding high-dose aspirin (300 to 325 mg/day) on the recurrence of any adenomas was available from four studies [12, 14, 15, 17]. For high-dose aspirin, we observed a statistically non-significant RRR of 10% (RR, 0.90 [95% CI 0.68 to 1.18]) for any adenomas with substantial heterogeneity (I^2^ = 78.2%); however, a significant reduction of 27% (RR, 0.73 [95% CI 0.56 to 0.94]) was observed for advanced adenomas, with no heterogeneity (I^2^ = 0%) (Fig. 3).

### Publication bias

In a meta-analysis with fewer studies (less than 10), the power of the asymmetrical tests is too low to distinguish the chance from real asymmetry [44]. Hence, publication bias could not be assessed in our analysis because the number of included studies was small.

### Adverse effects

The included studies reported data on bleeding events, peptic ulcers, dyspeptic symptoms, cardiovascular adverse events, stroke and colorectal cancers (See Additional file 1: Table S6, published online)*.* Serious adverse events were uncommon. However, the incidence of stroke was statistically significantly higher in the aspirin group than the control group (*p* = 0.007). Other adverse event rates were similar between aspirin and placebo groups.

### Trial sequential analyses

For aspirin in any dose, trial sequential analyses (TSA) for recurrent adenomas and advanced adenomas based on the information size adjusting for the presence of heterogeneity among all the 5 trials is shown in Additional file 1: Figures S3 and S4 (published online). We calculated TSA with α = 0.05 and power 80% and a requisite heterogeneity-adjusted information size based on the intervention effect on adenoma recurrence suggested by the low bias risk RCTs using a random-effects model (RRR of 17% for any adenomas and 2518 patients; RRR of 30% for advanced adenomas and 3223 patients). Since both the monitoring boundaries and information size surpassed with a cumulative Z-statistic above 1.96, this confirmed the firm evidence for a beneficial effect of aspirin on incidence of recurrent adenomas (See Additional file 1: Figure S3, published online). Although the number of patients included in the meta-analysis of advanced adenomas (*n* = 2950) did not exceed the required information size (*n* = 3223), the cumulative evidence is conclusive for a 30% reduction of recurrent advanced adenomas because it has crossed the monitoring boundary for statistical significance (See Additional file 1: Figure S4, published online).

We also conducted trial sequential analyses by similar method for low and high-dose aspirin on the incidence of recurrent adenomas and advanced adenomas (See Additional file 1: Figures S5-S7, published online). Since the required information size (*n* = 1125) surpassed and the cumulative z-curve crossed the monitoring boundary, TSA indicated a firm evidence to demonstrate a 20% relative reduction for low-dose aspirin on recurrent adenomas (See Additional file 1: Figure S5, published online). However, TSA indicated lack of firm evidence to demonstrate or reject a beneficial effect of 34% relative reduction for low-dose aspirin (See Additional file 1: Figure S6, published online) and 27% relative reduction for high-dose aspirin (See Additional file 1: Figure S7, published online) on recurrent advanced adenomas. We did not perform TSA for high-dose aspirin on the incidence of recurrent adenomas due to the substantial heterogeneity identified during meta-analysis (Fig. 3).

### GRADE summary of evidence for aspirin

GRADE summary of findings and strength of evidence for aspirin in reducing both adenoma and advanced adenoma recurrence is shown in Additional file 1: Table S7. Randomized trials without important limitations are rated high on the GRADE scale. Apart from one trial [17] there was no serious risk of bias in the trials. There was no serious inconsistency identified between trials. Apart from one [15], all the trials enrolled patients with history of adenoma; the remaining study enrolled patients with history of colorectal cancer. Moreover, interventions were delivered in different doses and the duration of follow-up varied among these studies (refer Table 1). Hence, we downgraded the rating because of questionable directness in the summary. The total sample size was limited and event rates were low in the case of incidence of recurrent advanced adenomas and we addressed this problem with trial sequential analysis. In context with the evidence from trial sequential analysis we chose not to downgrade on imprecision. Our application of GRADE-methodology led us to conclude that the accumulated evidence for aspirin at any dose or low dose is of moderate quality for adenoma prevention. For the effect on incidence of recurrent advanced adenomas, the evidence indicated low GRADE quality for low-dose aspirin.

### Effect of non-aspirin NSAIDs on incidence of recurrent colorectal adenomas

Characteristics of the included studies and study participants are shown in Table 2. Among three RCTs [28, 37, 38], all studies had low risks of bias in almost all criteria (See Additional file 1: Table S5 and Figure S8). In two RCTs [37, 38], the authors calculated the relative risk using data from both the 1-year and 3-year time points and did not report raw event data; hence, we pooled the relative risks from these two trials using inverse variance method. The pooled summary demonstrated statistically significant reductions in the incidence of recurrent adenomas and advanced adenomas over a 3-year follow-up (pooled relative risk, 0.66 [95% CI, 0.59 to 0.72] vs. 0.45 [CI, 0.33 to 0.57], respectively) for celecoxib 400 mg/day [28] (See Additional file 1: Figures S9 and S10). A similar protective effect was demonstrated by rofecoxib 25 mg/day for the prevention of recurrence of both adenomas (RR, 0.76 [0.69 to 0.83]) and advanced adenomas (RR, 0.56 [0.42 to 0.75]). The results from individual studies are summarized in Table 2. However, an increased risk for adverse cardiovascular outcomes associated with COX-inhibitors, as previously described [6, 45–47], represents a crucial drawback.

### Effect of NSAIDs withdrawal on incidence of recurrent adenomas: Post-trial follow-up results

Four post-trial studies [25–28] were available to investigate the effect of drugs withdrawal on recurrent adenoma incidence. Additional file 1: Table S4 describes the identified studies. Our study was restricted to subjects with or without adenomas detected during the intervention period and for whom colonoscopy findings were provided at the end of the post-trial observation period.

The post-trial follow-up results from studies are summarized in Additional file 1: Table S4. Two studies [26, 27] assessed all subjects who underwent colonoscopy approximately 2 years after treatment cessation with celecoxib, whether or not adenomas had been detected in them previously, demonstrated the absence of a protective effect after discontinuing regular use of celecoxib. Among these two studies [26, 27], one [26] demonstrate a significant increased risk of recurrent adenomas (RR, 1.48 [95%CI 1.19 to 1.83]) in all subjects after treatment cessation; a finding similar to the post-trial results (RR1.21 [95%CI 1.01 to 1.45]) of APPROVe study [28]. However, in a small study by Takayama et al. [43] does not demonstrated the absence of protective effect after 1 year in subjects who treated with non-aspirin NSAIDs for 2 months.

Follow-up of the Aspirin/Folate Polyp Prevention study demonstrated the extended chemopreventive effects of aspirin that were seen during the treatment period in all subjects who had been off study aspirin for 3 to 5 years and who continued the post-treatment use of aspirin and/or other NSAIDs [25]. We observed an apparent trend of strengthening of the chemopreventive effect associated with increased NSAID use during the post-trial period (Additional file 1: Table S4).

## Discussion

We identified five RCTs for aspirin and six for non-aspirin NSAIDs to update the effects on incidence of recurrent adenomas. All RCTs identified for aspirin were of good quality, with high compliance and generally with high follow-up rates, except one study [17]. However, apart from three trials for non-aspirin NSAIDs, others were associated with substantial risk of systematic errors. Hence we were only able to update the summary of effects of aspirin using all five randomized trials including the latest report of APACC trial [17] and a recently published study by Ishikawa et al. [16] Contrary to previous meta-analyses on aspirin [8–11, 23], there are some difference between their study and ours (See Additional file 1: Table S8, published online). We have assessed random errors in the meta-analysis and integrated the GRADE rating, thus expand the base for a well-founded judgment of the available evidence. Random errors consider as one of the major problems of unreliable findings due to meta-analyses [22, 48]. However, it has not previously been assessed in this field and may therefore contribute an important addition. Moreover, we addressed the effects of NSAIDs on the risk of recurrent adenomas after the withdrawal of the drug; a concern no reviews addressed previously.

Updated summary of effects of aspirin suggest that the regular use of aspirin (at any dose) lasting 2 to 4 years appears to reduce the incidence of recurrent colorectal adenomas with a pooled 17% RRR in patients with a previous history of colorectal cancer or adenomas. The reduction in the risk of recurrent advanced adenomas was more substantial with a pooled RRR of 30%. Our results remain largely the same as in the previous meta-analyses results [8–10]. Trial sequential analysis (TSA) indicated a firm evidence for a beneficial effect of aspirin on recurrent adenomas and advanced adenomas. Using GRADE-methodology we are led to conclude that the quality of the evidence is *moderate*.

Although aspirin at any dose seems to be an attractive choice for adenoma chemoprevention, doses those used for cardiovascular protection may provide an additional advantage as the balance of benefits and risks seems to be more favourable for low-dose aspirin [5, 18, 19]. Hence, we conducted a subgroup analysis to know whether the dose modifies the effect of aspirin on recurrent adenoma and advanced adenoma incidence. For low-dose aspirin, we have observed a significant 20% reduction of recurrent adenomas. TSA indicated a firm evidence for a beneficial effect of low-aspirin on recurrent adenomas. In contrast to the earlier meta-analyses [8, 9], however, with the inclusion of additional studies, low-dose aspirin demonstrated a statistically significant reduction in recurrent advanced adenomas. However, TSA indicated lack of firm evidence for this beneficial effect. An obvious reason for this discrepancy could be the lack of enough sample size as the required information size not reached to detect an intervention effect of this size as shown in TSA. The information size required to demonstrate or reject a 34% relative reduction of recurrent advanced adenomas with low-dose aspirin using 5% risk of type I error is 2547 patients (see Additional file 1: Figure S6, published online). This information size is far from reached with only 1178 patients randomized in three conducted trials of low-dose aspirin. More high quality randomized trials comparing low-dose aspirin versus placebo are still needed to conclude the evidence for low-dose aspirin on recurrent advanced adenomas.

The surprising lack of efficacy of the high dose aspirin and unusual dose response pattern as seen in the two multiple-dose trials (AFPPS and APACC trials) [12, 17] (Refer Table 1), together with substantial heterogeneity observed during meta-analysis (Fig. 3) prevents secure conclusion regarding the effect of high-dose aspirin on recurrent adenoma incidence.

COX-2 inhibitors (celecoxib 400–800 mg/day and rofecoxib 25 mg/day) seem to be highly effective in reducing the incidence of recurrent colorectal adenomas and advanced adenomas. However, due to the risk for gastrointestinal [49, 50] or cardiovascular [6, 10, 46, 47, 51, 52] harms associated with COX-2 inhibitors as shown in previous systematic reviews, does not appear to favour as a chemopreventive agent.

We observed no serious adverse events in terms of myocardial infarction, gastrointestinal bleeding, peptic ulcer, dyspepsia and colorectal cancer with the use of aspirin in any dose lasting 2 to 4 years in patients with a previous history of colorectal cancer or adenomas. We saw a higher rate of stroke among aspirin-treated participants, as previously reported by Cole et al. [8]. There is no clear explanation for these findings. Though, good quality RCTs on cardiovascular outcomes in patients without vascular disease informed that aspirin had no significant risk of ischemic stroke in men [53, 54], and may reduce this risk in women [53]. Moreover, high-quality evidence has shown that aspirin can decrease serious adverse events in patients at increased risk for cardiovascular disease [55]. However, a dose effect for aspirin was demonstrated with the risk for gastrointestinal toxicity and haemorrhagic stroke [5, 56, 57]. Use of low-dose aspirin in these individuals would results in positive cardiovascular effects, fewer adverse outcomes and they would get added benefit of fewer colorectal adenomas as shown in our analysis. However, additional studies on low-dose aspirin on advanced adenomas required to conclude the precise benefit of adenoma prevention.

We found the protective effect of aspirin on recurrent adenomas does not significantly reduce over time after treatment cessation [25]. A finding consistent with the observed chemoprevention of aspirin against colorectal cancer as previously shown by post-trial studies [58–60]. However, the greater protective effect of COX-2 inhibitors as shown in RCTs [28, 37, 38] did not persist during the post-treatment period; moreover, an increased risk of adenoma incidence was seen approximately 1–2 years after treatment cessation [26, 28]. This discrepancy may arise because of discontinuity of COX-2 inhibition or because of the cessation of alternative mechanisms independent of COX-2 inhibition as described previously [61]. However, the post-trial results of the Takayama et al. study [43], does not demonstrate an absence of protective effect after NSAIDs withdrawal. This could be due to the short treatment and follow-up periods (2 months and 1 year, respectively) of the Takayama et al. study compared to other post-trial studies [26, 27]. Confirmation of the increased adenoma incidence after the withdrawal of COX-2 inhibitors and determination of the cause will require further study.

Although we have updated information on the effects of aspirin and other NSAIDs on the incidence of recurrent adenomas using recently published and good quality RCTs, this analysis also has substantial limitations. First, the five RCTs included in this review of aspirin were similar but not identical with regard to follow-up and the dose; moreover the difference in population in the CALGB study [15] compared to others leads to the indirectness of evidence. Secondly, because the follow-up of the studies was not sufficiently long, we could not explore the long-term effects of aspirin on the recurrence of adenomas and the progression to cancer. The one study [17] with a longer duration showed that aspirin did not reduce adenoma recurrence. The obvious explanation for this discrepancy may be due to the small sample size and the substantial number of late dropouts. However, the absence of studies with similar or longer follow-up hampers the confirmation for our explanation. Third, because of the limited number of studies or insufficient sample size, we were not able to confirm the dose-response of aspirin on recurrent adenoma and advanced adenoma incidence. Fourth, we were not able to identify recent RCTs to update the knowledge of the effects of non-aspirin NSAIDs/COX-2 inhibitors on recurrent adenoma incidence. Finally, the quality and quantity of available evidence from post-trial results limit the findings on the effect of NSAIDs withdrawal on the incidence of recurrent adenomas.

## Conclusions

In summary, the available randomized trials suggest that aspirin and COX-2 inhibitors reduce the risk of recurrence of colorectal adenomas in patients with a previous history of colorectal cancer or adenomas. However, COX-2 inhibitors are associated with important cardiovascular events and gastrointestinal harms. Moreover, the protective effect of these agents does not persist and there may even be an increased incidence of recurrent adenomas after their withdrawal. Hence, aspirin seems to have a worthwhile role as a chemopreventive agent. The accumulated evidence for aspirin is associated with fewer risks of systematic errors as well as random errors. Thus, the risk of spurious findings for a beneficial effect of aspirin derived from the cumulative data on recurrent adenoma incidence is minimal. Incidence of recurrent colorectal adenomas was also reduced with low-dose aspirin. However, low-dose aspirin failed to show a conclusive protective effect on recurrent advanced adenomas. Since the balance of benefits to risk does favor low-dose aspirin, additional high quality randomized trials on low-dose aspirin required to confirm the precise benefit of recurrent adenoma prevention.

## Additional file

Additional file 1: Supporting Information for Online Publication. (DOCX 709 kb)

## Abbreviations

CI: Confidence interval
GRADE: the Grading of Recommendations, Assessment, Development and Evaluation
NSAIDs: Nonsteroidal anti-inflammatory drugs
PRISMA: Preferred reporting items for systematic reviews and meta-analyses
RCT: Randomized controlled trial
RR: Relative risk
RRR: Relative risk reduction
TSA: Trial sequential analysis
