# Supplementary material for: Effects of aspirin and non-aspirin nonsteroidal anti-inflammatory drugs on the incidence of recurrent colorectal adenomas: a systematic review with meta-analysis and trial sequential analysis of randomized clinical trials
Source: BMC Cancer. 2017 Nov 14;17:763. doi: 10.1186/s12885-017-3757-8 (PMC5686945; doi:10.1186/s12885-017-3757-8)
Supplement: Additional file 1: — Supporting Information for Online Publication. (DOCX 709 kb) [file 12885_2017_3757_MOESM1_ESM.docx]

**Additional file 1: (Supporting Information for Online Publication)**

**Effects of aspirin and non-aspirin nonsteroidal anti-inflammatory drugs on the incidence of recurrent colorectal adenomas: A systematic review and meta-analysis with trial sequential analysis of randomized clinical trials**

Sajesh K Veettil, ^1^ Kean Ghee Lim,^2^ Siew Mooi Ching,^3,4^ Surasak Saokaew,^5,6^ Pochamana Phisalprapa,^7^ Nathorn Chaiyakunapruk,^5,8,9,10*^

**Abbreviations**

GRADE: Grades of Recommendation, Assessment, Development and Evaluation; NSAIDs: nonsteroidal anti-inflammatory drugs; TSA: trial sequential analysis; NR: not reported; RR: relative risk

**Content**

| **Supplementary Tables** | | **Page** |
| --- | --- | --- |
|  | **Table S1.** Search strategy in MEDLINE | **3** |
|  | **Table S2.** GRADE Working Group grades of evidence | **4** |
|  | **Table S3.** Reason for exclusion of identified studies | **4-5** |
|  | **Table S4:** Effect of NSAIDs withdrawal on incidence of **recurrent** adenomas (Post-trail  follow-up results) | **6-7** |
|  | **Table S5.** Quality assessment of included trials | **8-10** |
|  | **Table S6.** Adverse events in all aspirin trials | **11** |
|  | **Table S7.** GRADE Summary of evidence | **12** |
|  | **Table S8.** Characteristics of meta-analyses: effectiveness of aspirin for the prevention of **recurrent**  colorectal adenomas | **13-15** |
| **Supplementary Figures** | |  |
|  | **Figure S1.** A flow of study selection | **16** |
|  | **Figure S2.** Summary (A) and individual (B) risk of bias (ROB) of all included studies on aspirin. | **17** |
|  | **Figure S3.** Trial sequential analysis assessing effect of aspirin any dose on  **recurrent** adenoma incidence | **18** |
|  | **Figure S4.** Trial sequential analysis assessing effect of aspirin any dose on **recurrent** advanced adenoma incidence. | **19** |
|  | **Figure S5.** Trial sequential analysis assessing effect of low-dose aspirin on **recurrent** adenoma incidence. | **20** |
|  | **Figure S6.** Trial sequential analysis assessing effect of low dose aspirin on **recurrent** advanced adenoma incidence. | **21** |
|  | **Figure S7.** Trial sequential analysis assessing effect of high dose aspirin on **recurrent** advanced adenoma incidence. | **22** |
|  | **Figure S8.** Summary (A) and individual (B) risk of bias (ROB) of all included  studies on non-aspirin NSAIDs. | **23** |
|  | **Figure S9.** Incidence of **recurrent** adenomas (COX-inhibitors versus  placebo) | **24** |
|  | **Figure S10.** Incidence of **recurrent** advanced adenomas (COX-inhibitors  versus placebo) | **24** |
|  | **Reference** | **25-26** |

**Table S1:** **Search strategy in MEDLINE**

| **Search strategy for parent study (Medline)** | |
| --- | --- |
| 1 | Terms for colorectal cancer or adenomas  (exp Colorectal Neoplasms/ OR exp Colonic Neoplasms/ OR exp Rectal Neoplasms/ OR exp Adenomatous Polyps/ OR exp Adenocarcinoma/ OR exp Intestinal Polyps/ OR exp Colonic Polyps/) OR ((colorectal cancer$.tw OR colorectal tumo$.tw OR colorectal neoplas$.tw OR colon cancer$.tw OR colon tumo$.tw OR colon neoplas$.tw OR colonic cancer$.tw OR colonic tumo$.tw OR colonic neoplas$.tw OR rectal cancer$.tw OR rectal tumo$.tw OR rectal neoplas$.tw OR rectum cancer$.tw OR rectum tumo$.tw OR rectum neoplas$.tw OR polyp$.tw OR adenoma$.tw OR adenomatous$.tw) OR (exp Adenoma/)) |
| 2 | Terms for NSAIDs and aspirin  (exp Anti-Inflammatory Agents, Non-Steroidal/ OR exp cyclooxygenase inhibitors/ OR exp  cyclooxygenase 2 inhibitors/ OR exp Aspirin/) OR (NSAID$.tw. OR Non-steroidal anti-inflammatory$.tw. OR Nonsteroidal anti-inflammatory$.tw. OR Non-steroidal antiinflammatory$.tw. OR Nonsteroidal antiinflammatory$.tw. OR Cyclo-oxygenase inhibitor$.tw. OR Cyclooxygenase inhibitor$.tw. OR Cyclooxygenase 1 inhibitor$.tw. OR Cyclooxygenase 2 inhibitor$.tw. OR COX-2 inhibitor$.tw. OR COX-2 selective inhibitor$.tw. OR COX-1 inhibitor$.tw. OR Coxib$.tw. OR Aspirin.af. OR Acetylsalicylic acid.tw.) |
| 3 | Terms for folic acid  (exp Folic Acid/ OR folate$.tw. OR folic$.tw. OR folic Acid.af.) |
| 4 | Terms for calcium  (exp Calcium, Dietary/ OR exp Calcium/ OR calcium.tw.) |
| 5 | Terms for vitamin D  (exp Cholecalciferol/ OR exp Ergocalciferols/ OR vitamin d.tw. OR Cholecalciferol$.tw. OR Ergocalciferol$.tw.) |
| 6 | Terms for antioxidants  (exp Antioxidants/ OR anti-oxidant$.tw. OR antioxidant$.tw. OR Selenium/ OR exp Vitamin A/ OR exp Carotenoids/ OR carotenoid$.tw. OR beta-carotene.tw. OR exp Ascorbic Acid/ OR vitamin c.tw. OR exp Vitamin E/ OR exp Tocopherols/ OR Tocotrienols/ OR alpha-tocopherol$.tw. OR tocopherol$.tw. OR tocotrienol$.tw.) |
| 7 | Terms for randomized controlled trial  (randomized controlled trial.pt. OR controlled clinical trial.pt. OR exp Clinical Trial/ OR Randomized controlled trials/ OR random allocation/ OR double blind method/ OR single blind method/ OR clinical trial.pt. OR placebos/ OR placebo$.ti,ab. OR random$.tw OR blind$.ti,ab.) |
| 8 | (1 AND (2 OR 3 OR 4 OR 5 OR 6) AND 7)  Limit 8 to (humans and yr="2008 - 2015") |
| 9 | Update the search: August 2015- September 2016 |

**Table S2:** GRADE Working Group grades of evidence*

| **Grading** | **Meaning** |
| --- | --- |
| High quality | We are very confident that the true effect lies close to that of the estimate of the effect |
| Moderate quality | We are moderately confident in the effect estimate: The true effect is likely to be close to the estimate of the effect, but there is a possibility that it is substantially different |
| Low quality | Our confidence in the effect estimate is limited: The true effect may be substantially different from the estimate of the effect |
| Very low quality | We have very little confidence in the effect estimate: The true effect is likely to be substantially different from the estimate of effect |

*The quality of evidence based on grading system can be rated down based on risk of bias (i.e., low risk, unclear, high risk), indirectness (i.e., measurement outcome such as death (direct outcome) or (indirect outcome)), imprecision (i.e., wide range of 95%CI), inconsistency (or heterogeneity) and/or publication bias, to levels of moderate, low and very low quality [1,2].

**Table S3: Reason for exclusion of identified studies**

| **Study** | **Reason for exclusion** |
| --- | --- |
| **Aspirin**- excluded studies | |
| Benamouzig 2003  (APACC) Study-1 year results) [3] | The primary outcomes of this study were defined as the proportion of subjects in whom at least one new adenoma was detected, the size of new adenomas, and the adenomatous polyp burden at the follow-up colonoscopy 1 and 4 years after enrolment. Since, the latest 4-year results of this study (Benamouzig 2012) available; we used the results from Benamouzig 2012 for our analysis. |
| HC Pommergaard 2016 [4] | Randomized, double-blind, placebo-controlled trial  Interventions and participants: mixture of 0.5 μg calcitriol, 75 mg acetylsalicylic acid, and 1250 mg calcium carbonate (n = 209), or placebo (n = 218)  Not separate arm for aspirin; calcium demonstrated some protective effect on adenoma recurrence; hence the results from this RCT cannot assign only to aspirin. |
| *Li ZY* et al 2011 [5] (Reference: *Li ZY, Gu JL, Zeng Z, Shi W (2011). Clinical study of aspirin in the prevention of recurrence of colorectal adenoma in the elderly. Chinese J Med Guide, 13, 89*) | We think that, this study is only a comparative clinical study NOT an RCT. We were not able to identify this study in our search. This study was identified from the reference list of Zhao et al review. As per their description, this paper retrieved from “Chinese biomedical literature service system (SinoMed) database”; it’s a Chinese study written in Chinese language; and graded as low quality. We searched “Chinese biomedical literature service system (SinoMed) database” to retrieve this article. Unfortunately, we were not able to identify the required information on this study. Hence, we communicated with the authors for getting full text/descriptions of this study. However, we didn’t received any reply. Moreover, the data obtained demonstrated in Zhao et al review for this study showed a control event rate (adenoma incidence) of 80% (n/N=39/49), which is too higher than what we saw in other trials (27% to a maximum of 47%). |
| **Non-aspirin NSAIDs**- excluded studies | |
| Meyskens 2008 [6] | Randomized Placebo-Controlled, Double-Blind  Trial Interventions and participants: Difluoromethylornithine (DFMO) 500mg and Sulindac 150 mg (n=191); Placebo (n=184)  No separate arm for sulindac. DFMO itself a chemo-preventive agent; hence the results from this RCT cannot assign only to sulindac. |
| Ladenheim 1995 [7] | Randomized placebo trial  Trial Interventions and participants: Sulindac 300mg/day (n=44); Placebo (n=40)  Primary outcome is percentage of patients for whom all polyps either disappeared or regressed. Incidence of adenoma not reported. |

**Table S4: Effect of NSAIDs withdrawal on incidence of recurrent adenomas (Post-trail follow-up results)**

| **Effect of NSAIDs withdrawal on incidence of recurrent adenomas (Post-trail follow-up results)** | | | | | | |
| --- | --- | --- | --- | --- | --- | --- |
| **Study;**  Author (year)  (Characteristics of population, outcomes and results of RCTs are given in table 1) | **Interventions** | **Duration of treatment** (Duration of follow-up colonoscopy) | **Number of subjects** (Treatment/Placebo), (n=total subjects); **number (%) of subjects excluded from main analyses** | **Baseline comparability** | **NSAIDs use during post-trial follow-up** | **Relative Risk (95% CI)** |
| **Follow-up of *Aspirin/Folate Polyp Prevention***  ***Study (AFPPS)*** [16]  Grau et al. (2009)- | Aspirin (Aspirin 81 mg/day or Aspirin 325 mg/day) versus  placebo | 3 years (≈3-5 years after end of the trial) | RCT: 749/372 (n=1121);  Post-trail follow up: 565/285 (n=850);  288 of 1121 (25.7%) excluded from final analysis.  (Of the 850 subjects who had post-treatment colonoscopy, 833 participants reported questionnaire about NSAIDs use) | Similar between groups | Among the 833 subjects, average use of NSAIDs ((aspirin (59%); ibuprofen (46%); naproxen (20%); coxibs (16%) :  <2 days/week: 589 (71%);  2 to <4 days/week: 96 (12%)  ≥4 days/week: 148(18%) | **Adjusted RR (95% CI);**  **aspirin (any dose)-**  **Incidence of adenoma**  Subjects using post-trail NSAIDs (≥4 days/week) - (0.67 [95% CI, 0.47 to 0.94])  Subjects using post-trail NSAIDs (<2 days/week) –(1.00 [95% CI, 0.80 to 1.24])  **Low dose aspirin –**  **Incidence of adenoma**  Subjects using post-trail NSAIDs (≥4 days/week) - (0.62 [95% CI, 0.39 to 0.98])  Subjects using post-trail NSAIDs (<2 days/week) –(1.01 [95% CI, 0.78 to 1.30])  **High dose aspirin –**  **Incidence of adenoma**  Subjects using post-trail NSAIDs (≥4 days/week) - (0.72[95% CI, 0.46 to 1.12])  Subjects using post-trail NSAIDs (<2 days/week) –(0.98 [95% CI, 0.76 to 1.27])  **Incidence of advanced adenoma**  Numbers for advanced adenomas were too small to draw confident conclusion |
| ***Follow-up of the Prevention of Colorectal Sporadic***  ***Adenomatous Polyps (Pre SAP) study*** [17]  Arber et al. (2011)- | Celecoxib 400 mg/day versus placebo | 3 years (2 years after end of the trial) | RCT: 933/628 (n=1561);  Post-trail follow up: 508/347 (n=855);  706 of 1561 (45.2%) excluded from final analysis. | Similar between groups | Low-dose aspirin (≤162.5mg every day or 325mg every other day) - compliance not reported.  Aspirin users: 120 (14%)  Non-aspirin users: 735 (86%) | **Incidence of adenoma**  All subjects- [1.48 (1.19 to 1.83)]  Subjects using post-trail NSAIDs - [2.19 (1.20 to 4.00)]  Subjects not using post-trail NSAIDs-[1.38 (1.09 to 1.74)]  **Incidence of advanced adenoma**  Not reported |
| **Follow-up of *the Adenoma Prevention with Celecoxib (APC) trial*** [18]  Bertagnolli et al. (2009) | Celecoxib 400 mg/day versus  celecoxib 800 mg/day versus  placebo | 3 years (≈2 years after end of the trial) | RCT: 685/671/679 (n=2035);  Post-trail follow up: 207/218/214 (n=639);  1396 of 2035 (68.5%) excluded from final analysis. | Similar between groups | Low-dose aspirin (≤325mg or 162.5mg every other day)- compliance not reported.  Aspirin users: 209 (32.7%)  Non-aspirin users: 430 (67.3%) | **Incidence of adenoma**  All subjects  celecoxib 400 mg/day - [1.09(0.85 to 1.38)]  celecoxib 800 mg/day - [1.11 (0.89 to1.41)]  Subjects using post-trail NSAIDs  celecoxib 400 mg/day - [1.51 (0.97 to 2.37)]  celecoxib 800 mg/day - [1.52 (0.98 to 2.37)]  Subjects not using post-trail NSAIDs  celecoxib 400 mg/day - [0.93 (0.7 to 1.3)]  celecoxib 800 mg/day - [0.97 (0.73 to 1.28)]  **Incidence of advanced adenoma**  All subjects  celecoxib 400 mg/day - [1.21 (0.56 to 2.65)]  celecoxib 800 mg/day - [1.95 (0.97 to 3.92)] |
| **Follow-up of *the Adenomatous Polyp PRevention On Vioxx (APPROVe)***  ***Trial*** [15]  Baron (2006) | Rofecoxib 25 mg/day versus  placebo | 3 years (1 year after end of the trial) | RCT: 1293/1277  (n=2570);  Post-trail follow up: 561/644(n=1205);  1365 of 2570 (53.1%) excluded from final analysis. | Not reported | Not reported | **Incidence of adenoma**  [1.21 (1.01 to 1.45)]  **Incidence of advanced adenoma**  Not reported |
| **Takayama 2011** [19] | Sulindac 300 mg/day;  Etodolac 400 mg/day;  Placebo | 2 months (1 year after the baseline examination) | Post-trail follow up:  Sulindac 300 mg/day (n=60);  Etodolac 400 mg/day (n=61);  Placebo (n=58)  171 of 179(4.5%) excluded from final analysis. | Similar between groups | Average compliance with medication was  92.7%, with similar compliance between arms. | **Relative Risk (95% CI) (only considered subjects with polypectomy)**  **Incidence of adenoma**  Sulindac – [0.39 (0.17–0.89)]  Etodolac – [0.96 (0.43–2.15)] |

**Table S5:** **Quality assessment of included trials**

| **Study (reference)** | **Random sequence generation** | **Allocation concealment** | **Blinding of participants and personnel** | **Blinding of outcome assessment** | **Incomplete outcome data** | **Selective reporting** | **Other bias** | **Judgement (Inclusion/Exclusion)** |
| --- | --- | --- | --- | --- | --- | --- | --- | --- |
| **Effect of aspirin on recurrent adenoma incidence** | | | | | | | | |
| **Baron2003 (AFPPS)** [8] | Computer generated; **low risk** | Central pharmacy; concealed; **low risk** | Double blind; blinding of participants and key study personnel ensured; **low risk** | Double blind; probably done; **low risk** | 37 of 1121 (3%) excluded from analysis as no-follow-up colonoscopy; missing data imputed; **low risk** | The study protocol is available and all of the study’s pre-specified (primary and secondary) outcomes that are of interest in the review have been reported; **low risk** | The study appears to be free of other sources of bias; **low risk** | Low risk of bias for all key domains; include the trial for final analysis |
| **Sandler 2003 (CALGB)** [9] | Probably done, but method not stated; baseline comparability similar; **low risk** | Assignment was made centrally by the CALGB Statistical  Centre; **low risk** | Double blind; identical-appearing placebo; **low risk** | Double blind; “endoscopy was not performed solely for the purposes of this study, but as part of the usual follow-up”- probably done; **low risk** | 118 of 635 (19%) excluded from analysis as no-follow-up colonoscopy; missing outcome data balanced in numbers across intervention groups, with similar reasons for missing data across groups; **low risk** | The study protocol is available and all of the study’s pre-specified (primary and secondary) outcomes that are of interest in the review have been reported; **low risk** | Enrolment of patients with colorectal cancer who had received curative treatment (surgical resection) may affect the incidence rate of adenomas. Appears to be free from other bias; **unclear [**As per the our protocol for systematic review, the participants defined as “patients with history of colorectal cancer or adenomas (increased risk individuals)”; Participants in this study comes under the defined population as per protocol] | Plausible bias due to the difference in study population between trails unlikely to seriously alter the results of meta-analysis; include the trial for final analysis |
| **Logan 2008**  **(ukCAP)** [10] | Computer generated; **low risk** | Central pharmacy; **low risk** | Double blind; “The researchers and all clinical staff involved with patient recruitment were blind to this treatment-allocation schedule”; **low risk** | Double blind; investigators were blind to the treatment; **low risk** | 92 of 945 (10%) excluded from analysis as no-follow-up colonoscopy; missing outcome data balanced in numbers across intervention groups, with similar reasons for missing data across groups; **low risk** | The study protocol is available and all of the study’s pre-specified outcomes that are of interest in the review have been reported; **low risk** | The study appears to be free of other sources of bias; **low risk** | Low risk of bias for all key domains; include the trial for final analysis |
| **Benamouzig 2012 (APACC)** [11] | computer-assisted method; not clear, but reviewer think probably done; **low risk** | Central pharmacy; **low risk** | “Patients, staff in the APACC Coordination Centre and study investigators were unaware of treatment assignment” – **low risk** | Investigators were blind to the treatment; **low risk** | 87 of 272 (32%) excluded from analysis as no-follow-up colonoscopy at year 4; **unclear** [Although drop-out rate is high, missing outcome data balanced in numbers across intervention groups, with similar reasons for missing data across groups. Reviewers think that, plausible bias unlikely to seriously alter the results] | The study protocol is available and all of the study’s pre-specified outcomes that are of interest in the review have been reported; **low risk** | Possibility of lack of statistical power due to 32% exclusion rate ; **unclear**  [missing outcome data balanced in numbers across intervention groups, with similar reasons for missing data across groups; Reviewers think that, plausible bias unlikely to seriously alter the results] | This is the most update results from APACC trail and the only trail with a long duration of follow-up of 4 years; hence review authors agree to use the most updated results (Not 1- year results from APACC study – Benamouzig 2003).  Plausible bias due to the large attrition rate unlikely to seriously alter the results of meta-analysis; include the trial for final analysis |
| **Ishikawa 2014** [12] | Computer-aided system; baseline comparability similar; reviewers believe randomization probably done adequately; **low risk** | Central allocation through Medical Research Support website; reviewers believe adequate concealment of allocation probably done; ; **low risk** | Stated double blind; but blinding was not clear for personnel involved in the trail; **unclear** | Stated “double-blinded (both subjects and investigators)” - probably done; **low risk** | 78 of 389 (20%) excluded from analysis as no-follow-up colonoscopy; missing outcome data balanced in numbers across intervention groups, with similar reasons for missing data across groups; **low risk** | The study protocol is available and all of the study’s pre-specified outcomes that are of interest in the review have been reported; **low risk** | The study appears to be free of other sources of bias; **low risk** | Plausible bias unlikely to seriously alter the results; include the trial for final analysis |
| **Effect of non-aspirin NSAIDs on recurrent adenoma incidence** | | | | | | | | |
| **Arber 2006 (Pre SAP study)** [13] | Method not clear; baseline comparability similar; reviewers believe randomization probably done adequately; **low risk** | Central pharmacy; **low risk** | Double blind; blinding of participants and key study personnel ensured; **low risk** | Stated “all polyps removed during  colonoscopies were submitted to local and central pathology laboratories for blinded review”; probably done; low risk | 164 of 1561 (11%) excluded from analysis as no follow-up colonoscopy at year 1 or year 3; with similar reasons for missing data across groups; low risk | Trail stopped early because of increased cardio vascular risks; all of the study’s pre-specified outcomes that are of interest in the review have been reported; low risk | The study appears to be free of other sources of bias; low risk | Low risk of bias for all key domains. Authors calculated the relative risk using data from both the 1-year and 3-year time points and not reported raw event data; hence not possible to do the statistical pooling of the results. |
| **Bertagnolli 2006**  **(APC trial)** [14] | Method not clear; baseline comparability similar; reviewers believe randomization probably done adequately; **low risk** | Central pharmacy; **low risk** | Stated “each capsule was identical in appearance”; Blinding of participants and key study personnel probably done; **low risk** | Stated “central study pathologist examined in a blinded fashion all polyps removed during these colonoscopies’; probably done; **low risk** | 213 of 2035 (10%) excluded from analysis as no follow-up colonoscopy at year 1 or year 3; with similar reasons for missing data across groups; **low risk** | Trail stopped early because of increased cardio vascular risks; all of the study’s pre-specified outcomes that are of interest in the review have been reported; **low risk** | The study appears to be free of other sources of bias; **low risk** | Low risk of bias for all key domains. Authors calculated the relative risk using data from both the 1-year and 3-year time points and not reported raw event data; hence not possible to do the statistical pooling of the results. |
| **Baron 2006**  **(APPROVe**  **Trial)** [15] | Stated “computer-derived randomization”; probably done adequately; **low risk** | Not stated, Probably done, **low risk** | Double blind study stated” Patients, investigators, and study monitoring staff were blinded to treatment allocations”; **low risk** | Stated ”investigators were blinded to treatment allocations”; **low risk** | 194 of 2570 (8%) excluded from analysis as no follow-up colonoscopy; ; **low risk** | Trail stopped early because of increased cardio vascular risks; all of the study’s pre-specified outcomes that are of interest in the review have been reported; **low risk** | The study appears to be free of other sources of bias; **low risk** | Low risk of bias for all key domains. |

**Table S6: Adverse events in all trials (aspirin versus placebo)**

| Adverse events | **Baron2003** [8] | | **Sandler 2003** [9] | | **Logan 2008** [10] | | **Benamouzig 2012** [11] | | **Ishikawa 2014** [12] | | **P*** |
| --- | --- | --- | --- | --- | --- | --- | --- | --- | --- | --- | --- |
|  | Aspirin 81mg/325mg (n=749) | Placebo (n=372) | Aspirin 325 mg (n=317) | Placebo (n=318) | Aspirin 300mg (n=472) | Placebo (n=467) | Aspirin  160 mg/300mg (n=140) | Placebo (n=132) | Aspirin 100mg (n=191) | Placebo (n=198) |  |
| Myocardial infarction | 7 | 1 | 7 deaths due to cardio-vascular causes | 5 deaths due to cardio-vascular causes | 1 | 2 | 0 | 1 | NR | NR | 0.56 |
| Stroke | 7 | 0 |  |  | 2 | 0 | 1 | 0 | NR | NR | 0007 |
| Coronary revascularization / Vascular events requiring aspirin | 8 | 4 |  |  | 7 | 9 |  |  | NR | NR | 0.54 |
| Bleeding (major/minor) | 6 (major) | 3 (major) | Two grade 4 adverse  events and Four grade 3 adverse events | Two grade 4 adverse events  and four grade 3 adverse events | 5 (NR) | 5(NR) | 24 (minor) | 20  (minor) | NR | NR | 0.73 |
| Peptic ulcer | NR | NR |  |  | 3 | 1 | NR | NR | NR | NR | 0.32 |
| Any dyspeptic symptoms | NR | NR |  |  | 108 | 116 | NR | NR | NR | NR | 0.48 |
| Colorectal cancer | 5 | 1 | 4 | 6 | 3 | 7 | NR | NR | 2 | 2 | 0.29 |
| NR: not reported; major bleeding: bleeding leading to hospitalization or surgical intervention; p*: values (two-sided) are from chi-squared test comparing aspirin vs placebo in any dose. | | | | | | | | | | | |

**Table S7:** **GRADE Summary of evidence**

| **Quality assessment** | | | | | | | | **№ of patients** | | **Effect** | | **Quality** | **Importance** |
| --- | --- | --- | --- | --- | --- | --- | --- | --- | --- | --- | --- | --- | --- |
| **№ of studies** | **Study design** | | **Risk of bias** | **Inconsistency** | **Indirectness** | **Imprecision** | **Other considerations** | **Aspirin in any dose** | **placebo** | **Relative (95% CI)** | **Absolute (95% CI)** |  |  |
| **Aspirin at any dose - Incidence of recurrent adenomas (follow up: range 2 year to 4 years)** | | | | | | | | | | | | | |
| 5 | randomised trials | not serious ^a^ | | not serious ^b^ | serious ^c^ | not serious ^d^ | none | 540/1668 (32.4%) | 468/1282 (36.5%) | **RR 0.83** (0.73 to 0.94) | **62 fewer per 1,000** (from 22 fewer to 99 fewer) | ⨁⨁⨁◯ MODERATE | IMPORTANT |
| **Aspirin at any dose - Incidence of recurrent advanced adenomas (follow up: range 2 year to 4 years)** | | | | | | | | | | | | | |
| 5 | randomised trials | not serious ^a^ | | not serious ^b^ | serious ^c^ | not serious ^d^ | none | 125/1668 (7.5%) | 128/1282 (10.0%) | **RR 0.70** (0.55 to 0.88) | **30 fewer per 1,000** (from 12 fewer to 45 fewer) | ⨁⨁⨁◯ MODERATE | IMPORTANT |
| **Low-dose aspirin - Incidence of recurrent adenomas (follow up: range 2 year to 4 years)** | | | | | | | | | | | | | |
| 3 | randomised trials | not serious ^a^ | | not serious ^e^ | serious ^f^ | not serious ^g^ | none | 211/573 (36.8%) | 277/605 (45.8%) | **RR 0.80** (0.70 to 0.92) | **92 fewer per 1,000** (from 37 fewer to 137 fewer) | ⨁⨁⨁◯ MODERATE | IMPORTANT |
| **Low-dose aspirin - Incidence of recurrent advanced adenomas (follow up: range 2 year to 4 years)** | | | | | | | | | | | | | |
| 3 | randomised trials | not serious ^a^ | | not serious ^e^ | serious ^f^ | serious ^h^ | none | 35/573 (6.1%) | 56/605 (9.3%) | **RR 0.66** (0.44 to 0.99) | **31 fewer per 1,000** (from 1 fewer to 52 fewer) | ⨁⨁◯◯ LOW | IMPORTANT |

**CI:** Confidence interval; **RR:** Risk ratio

^a^ Quality assessments of identified RCTs were described in Table S4.

^b^ Inconsistency explained by I^2^ statistic (low-moderate heterogeneity)

^c^ All studies except sandler et al. enrolled patients with a history of colorectal cancer; while other studies enrolled patients with history of adenoma; Interventions delivered in different doses (higher (300-325mg) or lower dose (80-160mg)) (Refer table 1); Time difference in outcome measures (2 to 4 years) (Refer table 1)

^d^ We addressed this problem with Trial Sequential Analysis (refer Figures S3 and S4).

^e^ No heterogeneity identified (I^2^ =0%)

^f^ Time difference in outcome measures (2 to 4 years)

^g^ Trial Sequential Analysis: the optimal information size criterion is achieved (refer Figure S5)

^h^ Trial Sequential Analysis: the optimal information size criterion is not achieved (refer Figure S6)

**Table S8:** Characteristics of meta-analyses: effectiveness of aspirin for the prevention of recurrent colorectal adenomas

| **Author, Year** | **Included RCTs** *(Refer table 1 for particulars of RCTs)* | **Comparison** | **Summary of meta-analyses (outcome: Incidence of recurrent colorectal adenomas)** |
| --- | --- | --- | --- |
| Cooper et al., 2010 [20] | - ukCAP trial - AFPPS - CAPS - APACC (1-year results) | Aspirin versus no aspirin | ***Any adenoma-*** Random effects, RR 0.79 (95% CI 0.68 to 0.92), with a moderate level of statistical heterogeneity (I^2^ = 34%)  ***Advanced adenoma-*** Random effects, RR 0.66 (95% CI 0.51 to 0.84), with no heterogeneity (I^2^ = 0%)  **Quality assessment:** Criteria based on recommendations from the centre for reviews and dissemination  **Trail sequential analysis:** not reported  **GRADE rating:** not reported |
| Cole et al., 2009 [21] | - ukCAP trial - AFPPS - CAPS - APACC* (results at 1 or 4 years) | Aspirin versus no aspirin | ***Any adenoma-*** Random effects, RR 0.83 (95% CI 0.72 to 0.96), with a moderate level of statistical heterogeneity (I^2^ = 41.5%)  ***Advanced adenoma-*** Random effects, RR 0.72 (95% CI 0.57 to 0.90), with no heterogeneity (I^2^ = 0%)  **Sub-group analysis:** Effect of high and low dose aspirin reported  **Quality assessment:** not reported  **Trail sequential analysis:** not reported  **GRADE rating:** not reported |
| Gao et al., 2009 [22] | - ukCAP trial - AFPPS - APACC (1-year results) | Aspirin versus no aspirin | ***Any adenoma-*** Fixed effects, RR 0.84 (95% CI 0.75–0.94). The test for heterogeneity between the studies was not statistically significant (P = 0.503)  ***Advanced adenoma-*** Fixed effects, RR 0.65 (95% CI 0.51–0.84). The test for heterogeneity between the studies was not statistically significant (P = 0.817)  **Sub-group analysis:** Effect of high and low dose aspirin reported  **Quality assessment:** Jadad score  **Trail sequential analysis:** not reported  **GRADE rating:** not reported |
| Wang et al., 2015 [23] | - APACC (1-year results) - AFPPS - ukCAP - APACC-2012 (4-year results) - Pre SAP study (both RCT and post-trial results were used) - APC trial (both RCT and post-trial results were used) - APPROVe Trial (both RCT and post-trial results were used) | Non-steroidal  anti-inflammatory drugs (NSAIDs) versus placebo (aspirin and other non-aspirin NSAIDs not analysed separately) | ***Incidence of adenoma and advanced adenoma:*** Results were categorized by the duration of follow-up. Results demonstrated that NSAIDs were associated with a significant decrease in adenoma recurrence at 1 and 3 years, but not beyond 3 years of follow-up.  In this review, authors combined the trials of both aspirin and COX-inhibitors. In their meta-analysis, post-trial results of Pre SAP study and APC trial (5 year analysis after 2 year treatment cessation) were combined with 4 year result of APACC trial (there is no treatment cessation).  **Quality assessment:** Jadad score  **Trail sequential analysis:** not reported  **GRADE rating:** not reported |
| Zhao et al., 2016 [24] | - ukCAP trial - AFPPS - CAPS - APACC (2012) (4-year results) - Ishikawa (2014) - ZHY Yang et al (2011)** | Aspirin alone versus placebo | ***Incidence of adenoma and advanced adenoma:*** Results were categorized by the duration of follow-up and population. Summary: Aspirin is associated with a remarkable decrease in the recurrence of any adenoma and advanced adenomas without concerning the dose of aspirin.  **Quality assessment:** Jadad score  **Trail sequential analysis:** not reported  **GRADE rating:** not reported |
| Present analysis | **Aspirin**   - ukCAP trial - AFPPS - CAPS - APACC (2012) (4-year results) - Ishikawa 2014 | Aspirin alone versus placebo | ***Incidence of adenoma and advanced adenoma:*** Results were categorized by aspirin at any dose, low-dose and high dose.  **Aspirin at any dose:**  *Any adenoma****-*** Random effects, RR 0.83 (95% CI 0.73 to 0.94); with a moderate level of statistical heterogeneity (I^2^ = 29.8%)  *Advanced adenoma****-*** Random effects, RR 0.70 (95% CI 0.55 to 0.88); with no heterogeneity (I^2^ = 0%)  **Sub-group analysis:** Effect of high and low dose aspirin reported  **Aspirin at low-dose:**  *Any adenoma****-*** Random effects, RR 0.80 (95% CI 0.70 to 0.92); with a moderate level of statistical heterogeneity (I^2^ = 0%)  **Trail sequential analysis:**  The cumulative evidence is conclusive for the above results.  **GRADE rating:** The accumulated evidence is of moderate quality. |
|  | **Non-aspirin NSAIDs**   - Pre SAP study - APC trial - APPROVe Trial | COX-2 inhibitors versus placebo | **Celecoxib 400mg/day versus placebo (inverse variance method)**  *Any adenoma- RR 0.66 (95% CI, 0.59 to 0.72)*  *Advanced adenoma- RR 0.45 (CI, 0.33 to 0.57)*  **Trail sequential analysis:**  no (required data for TSA not available)  **GRADE rating:** no |
|  | **Post-trial studies**   - Grau et al. (2009) [**Follow-up of *spirin/Folate Polyp Prevention Study (AFPPS)]*** - Arber et al. (2011) [***Follow-up of the Prevention of Colorectal Sporadic Adenomatous Polyps (Pre SAP) study]*** - Bertagnolli et al. (2009) [**Follow-up of *the Adenoma Prevention with Celecoxib (APC) trial]*** - Baron et al. (2006) ***[Follow-up of the Adenomatous Polyp PRevention On Vioxx (APPROVe) Trial]*** - Takayama 2011 | NSAIDs versus placebo ( Post-trial effects on adenomas) | Post-trial results were presented separately. A narrative overview of the findings of included studies were presented with tabular summaries of extracted data. |
| *Cole et al. used the unpublished 4-year data (incidence at 1 or 4 years) from the APACC trial. While in the present analysis, we used the published results at 4 years from APACC trial.  **ZHY Yang et al: Reference: Li ZY, Gu JL, Zeng Z, Shi W (2011). Clinical study of aspirin in the prevention of recurrence of colorectal adenoma in the elderly. Chinese J Med Guide, 13, 89 (Chinese paper). | | | |

**Figure S1:** A flow of study selection

Records excluded
(n =3840)

Full-text articles assessed for eligibility
(n = 145)

Records screened
(n =3985)

Records after duplicates removed
(n =3985)

Additional records identified through other sources
(n = 120)

Records identified through database searching
(n = 4989)

Identification

Full-text articles excluded, with reasons (As per parent study protocol) (n = 70)

Not an eligible study design (n=26)

Not an eligible population (n=2)

Not an eligible intervention (n=7)

Not an eligible outcome (n=32)

Duplicate (n=3)

Studies included in the present study (Aspirin & non-aspirin NSAIDS versus placebo)
(RCTs, n = 13; Post-trail follow-up results, n=4)

Among 13 RCTs, 5 were excluded with reason (refer appendix Table 3)

Included

Eligibility

Screening

Since Rofecoxib already withdrawn from the market, it was excluded in the parent study as per protocol (Network meta-analysis). However, in the present study we have included one RCT on rofecoxib to summarize the effect of non-aspirin NSAIDs.

Studies included in the systematic review of all interventions (Parent study)
(n = 75)

**Figure S2: Summary (A) and individual (B) risk of bias (ROB) of all included studies on aspirin.**

(A)


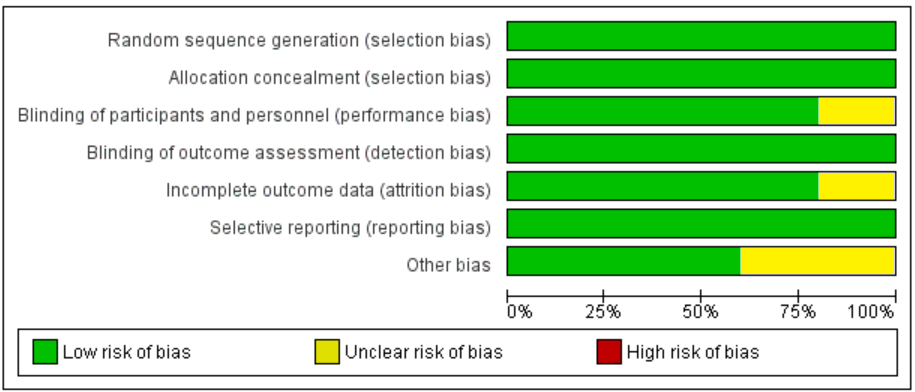


(B)


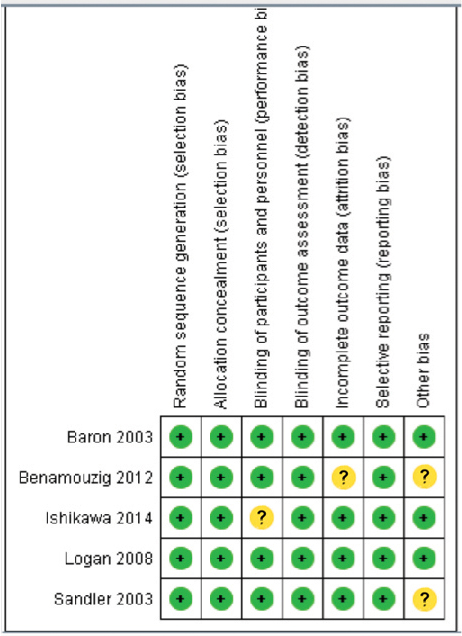


Quality assessment of included trials presented in Table S4

**Figure S3.** Trial sequential analysis assessing effect of aspirin any dose

on recurrent adenoma incidence


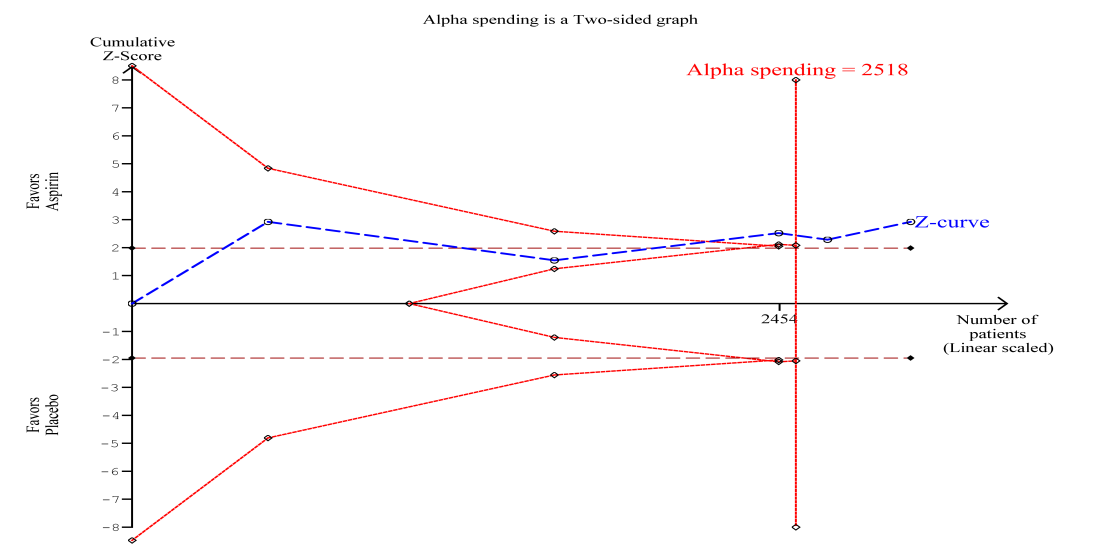


| **Trial sequential analysis assessing effect of aspirin any dose on adenoma incidence.**  TSA were calculated with type 1 error of 5% and type II error of 20% and a required heterogeneity-adjusted information size (n= 2518) based on the intervention effect suggested by the included trails using random-effects model for aspirin any dose (RRR of 17.35% (low-bias risk trail estimate)) and control group event proportion of 40% (median proportion of incidence of adenomas in the control group) (figure 1). Since both the monitoring boundaries and information size surpassed with a cumulative Z-statistic above 1.96, thereby confirming the firm evidence for a beneficial effect of aspirin on adenoma incidence. |
| --- |

**Figure S4.** Trial sequential analysis assessing effect of aspirin any dose on recurrent advanced adenoma incidence.

**
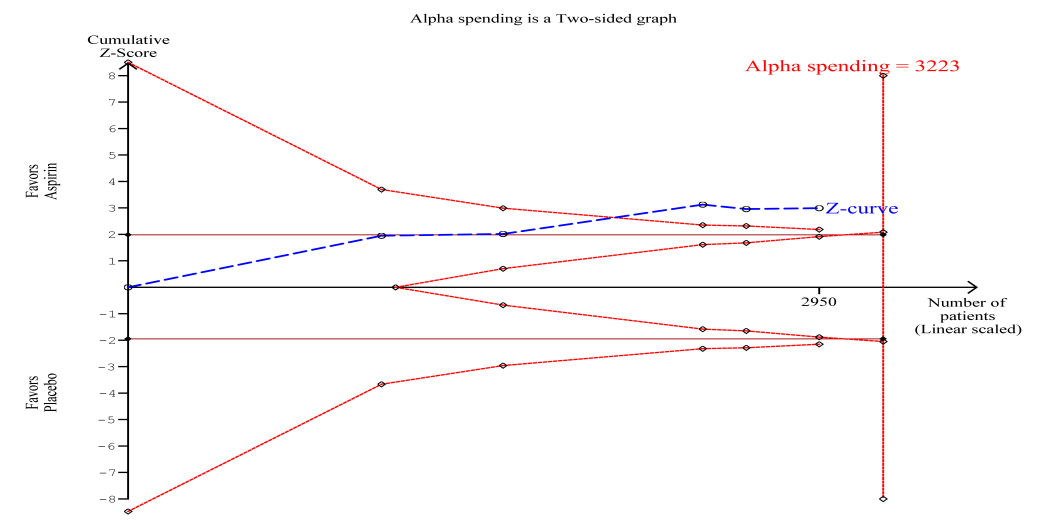
**

| **Trial sequential analysis assessing effect of aspirin any dose on advanced adenoma incidence.**  TSA were calculated with type 1 error of 5% and type II error of 20% and a required information size (n= 3223) based on the intervention effect suggested by the included trails using random-effects model for aspirin any dose (RRR of 30.24 % (low-bias risk trail estimate)) and control group event proportion of 8.4% (median proportion of incidence of advanced adenomas in the control group) (figure 2). Although the number of patients included in the meta-analysis did not exceed the required information size, the cumulative evidence is conclusive for 30% reduction of advanced adenomas because it has crossed the monitoring boundary for statistical significance. |
| --- |

**Figure S5.** Trial sequential analysis assessing effect of low-dose aspirin

on recurrent adenoma incidence.


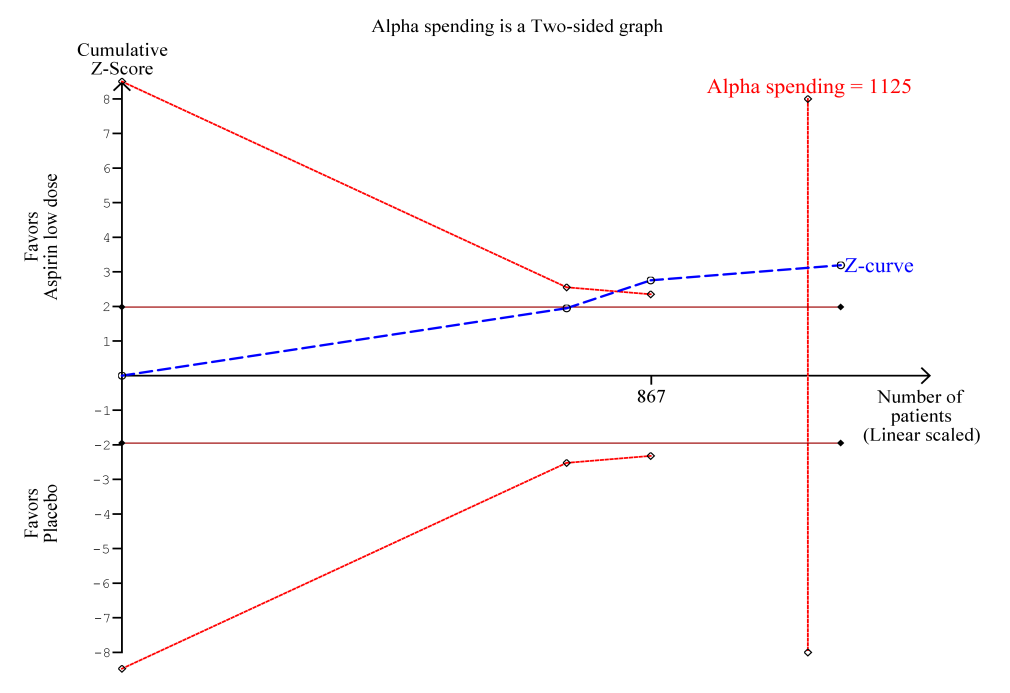


| **Figure 5. Trial sequential analysis assessing effect of low dose aspirin on recurrent adenoma incidence.**  The information size required to demonstrate or reject a 20% relative reduction (low-bias risk trail estimate) based on an assumption of 40% of control group event proportion (median proportion of incidence of recurrent adenomas in the control group) with type 1 error of 5% and type II error of 20% is 1125 patients. The number of patients included in the meta-analysis surpassed the information size and the cumulative z-curve crossed the monitoring boundary constructed for a required information size; hence, we are able to infer that low dose aspirin superior to the control in preventing recurrent adenoma incidence. |
| --- |

**Figure S6.** Trial sequential analysis assessing effect of low-dose aspirin

on recurrent advanced adenoma incidence.


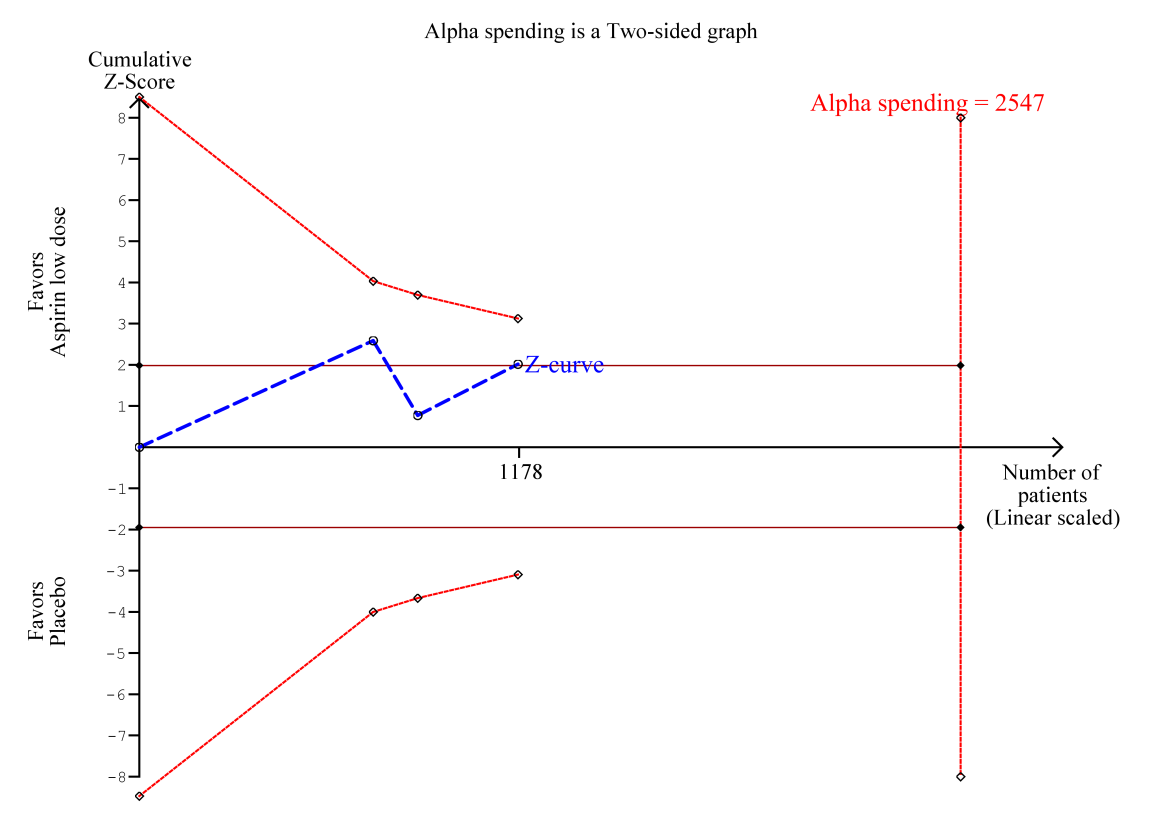


| **Figure 6. Trial sequential analysis assessing effect of low dose aspirin on recurrent advanced adenoma incidence.**  The information size required to demonstrate or reject a 34% relative reduction (low-bias risk trail estimate) based on an assumption of 8.4% of control group event proportion (median proportion of incidence of recurrent adenomas in the control group) with type 1 error of 5% and type II error of 20% is 2547 patients. Since the trial sequential monitoring boundary was not surpassed and the required information size not reached, TSA indicated lack of firm evidence for a beneficial effect of 34% RRR for low dose of aspirin on recurrent advanced adenomas. |
| --- |

**Figure S7.** Trial sequential analysis assessing effect of high-dose

aspirin on recurrent advanced adenoma incidence.


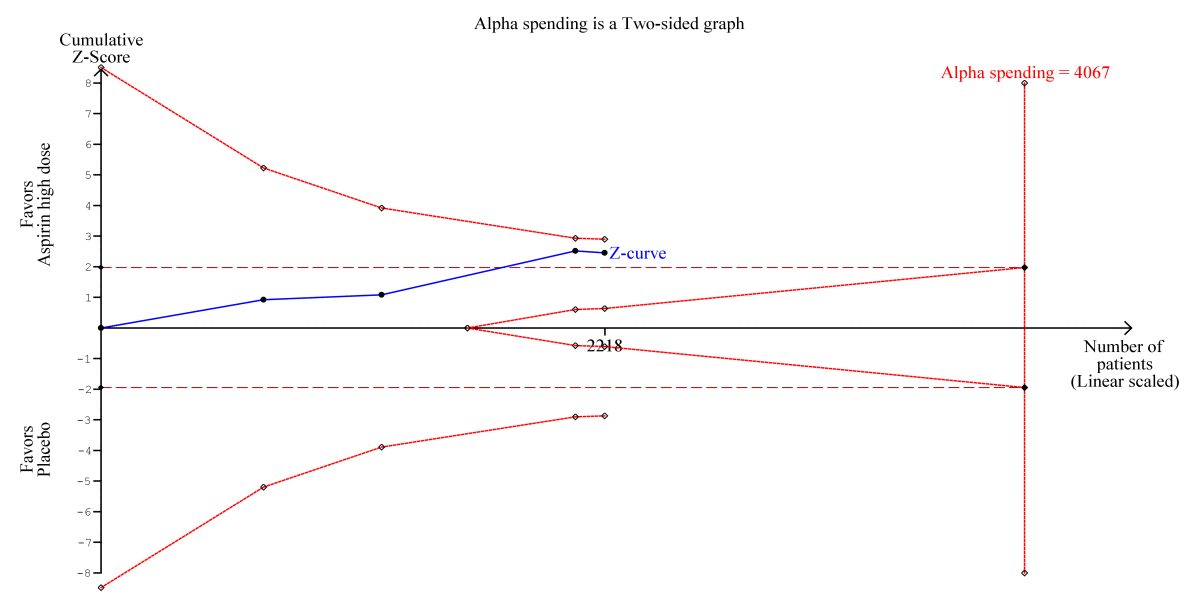


| **Figure 7. Trial sequential analysis assessing effect of high dose aspirin on recurrent advanced adenoma incidence.**  To determine the required sample size we assumed a 8.4% recurrent advanced adenoma incidence in the control group and a relative risk reduction of 27% (recurrent adenoma incidence and relative risk reduction among trials with low bias risk in our meta-analysis). Our calculations showed that we would need a sample of 4067 (with 80% power and α 0.05) to detect a plausible treatment effect for high dose aspirin on recurrent advanced adenoma incidence, corresponding to a relative risk reduction of 27%. Currently 2218 patients have been randomized and no trial sequential monitoring boundary crossed, indicating that the cumulative evidence is inconclusive for a 27% relative risk reduction. |
| --- |

**Figure S8.** Summary (A) and individual (B) risk of bias (ROB) of all

Included studies on non-aspirin NSAIDs.

(A)


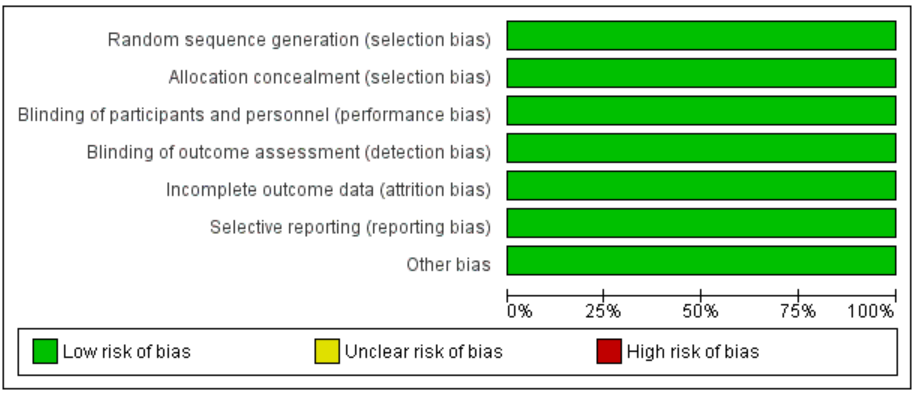


(B)


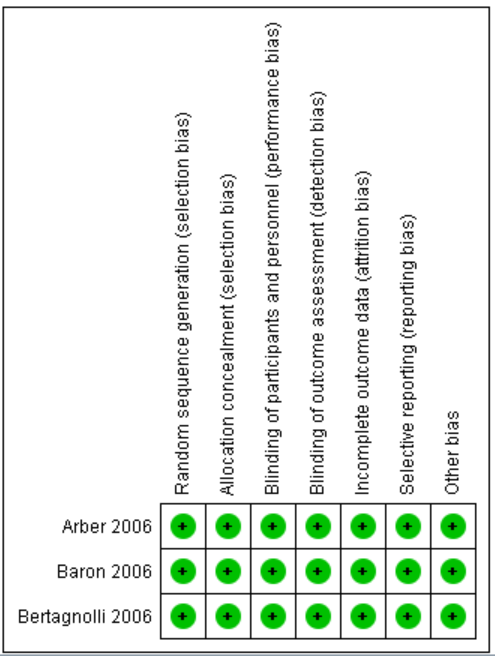


Quality assessment of included trials presented in Table S4

**Figure S9.** Incidence of recurrent adenomas: COX-2 inhibitors versus

placebo

*This analysis is based on reported relative risks rather than on raw event data, because the study authors calculated the*

*relative risk using data from both the 1-year and 3-year time points.*

**Figure S10.** Incidence of recurrent advanced adenomas: COX-2

inhibitors versus placebo

*This analysis is based on reported relative risks rather than on raw event data, because the study authors calculated the*

*relative risk using data from both the 1-year and 3-year time points.*

Reference

1. Atkins D, Best D, Briss PA, Eccles M, Falck-Ytter Y, Flottorp S, et al. Grading quality of evidence and strength of recommendations. BMJ. 2004;328:1490.

2. Guyatt GH, Oxman AD, Vist GE, Kunz R, Falck-Ytter Y, Alonso-Coello P, et al. GRADE: an emerging consensus on rating quality of evidence and strength of recommendations. BMJ. 2008;336:924–6.

3. Benamouzig R, Deyra J, Martin A, Girard B, Jullian E, Piednoir B, et al. Daily soluble aspirin and prevention of colorectal adenoma recurrence: one-year results of the APACC trial. Gastroenterology. 2003;125:328–36.

4. Pommergaard H-C, Burcharth J, Rosenberg J, Raskov H. Aspirin, Calcitriol, and Calcium Do Not Prevent Adenoma Recurrence in a Randomized Controlled Trial. Gastroenterology. 2016;150:114–22.

5. Li ZY, Gu JL, Zeng Z, Shi W (2011). Clinical study of aspirin in the prevention of recurrence of colorectal adenoma in the elderly. Chinese J Med Guide, 13, 89, 2011.

6. Meyskens FL, McLaren CE, Pelot D, Fujikawa-Brooks S, Carpenter PM, Hawk E, et al. Difluoromethylornithine plus sulindac for the prevention of sporadic colorectal adenomas: a randomized placebo-controlled, double-blind trial. Cancer Prev. Res. Phila. Pa. 2008;1:32–8.

7. Ladenheim J, Garcia G, Titzer D, Herzenberg H, Lavori P, Edson R, et al. Effect of sulindac on sporadic colonic polyps. Gastroenterology. 1995;108:1083–7.

8. Baron JA, Cole BF, Sandler RS, Haile RW, Ahnen D, Bresalier R, et al. A Randomized Trial of Aspirin to Prevent Colorectal Adenomas. N. Engl. J. Med. 2003;348:891–9.

9. Sandler RS, Halabi S, Baron JA, Budinger S, Paskett E, Keresztes R, et al. A Randomized Trial of Aspirin to Prevent Colorectal Adenomas in Patients with Previous Colorectal Cancer. N. Engl. J. Med. 2003;348:883–90.

10. Logan RFA, Grainge MJ, Shepherd VC, Armitage NC, Muir KR, ukCAP Trial Group. Aspirin and folic acid for the prevention of recurrent colorectal adenomas. Gastroenterology. 2008;134:29–38.

11. Benamouzig R, Uzzan B, Deyra J, Martin A, Girard B, Little J, et al. Prevention by daily soluble aspirin of colorectal adenoma recurrence: 4-year results of the APACC randomised trial. Gut. 2012;61:255–61.

12. Ishikawa H, Mutoh M, Suzuki S, Tokudome S, Saida Y, Abe T, et al. The preventive effects of low-dose enteric-coated aspirin tablets on the development of colorectal tumours in Asian patients: a randomised trial. Gut. 2014;63:1755–9.

13. Arber N, Eagle CJ, Spicak J, Rácz I, Dite P, Hajer J, et al. Celecoxib for the Prevention of Colorectal Adenomatous Polyps. N. Engl. J. Med. 2006;355:885–95.

14. Bertagnolli MM, Eagle CJ, Zauber AG, Redston M, Solomon SD, Kim K, et al. Celecoxib for the Prevention of Sporadic Colorectal Adenomas. N. Engl. J. Med. 2006;355:873–84.

15. Baron JA, Sandler RS, Bresalier RS, Quan H, Riddell R, Lanas A, et al. A randomized trial of rofecoxib for the chemoprevention of colorectal adenomas. Gastroenterology. 2006;131:1674–82.

16. Grau MV, Sandler RS, McKeown-Eyssen G, Bresalier RS, Haile RW, Barry EL, et al. Nonsteroidal anti-inflammatory drug use after 3 years of aspirin use and colorectal adenoma risk: observational follow-up of a randomized study. J. Natl. Cancer Inst. 2009;101:267–76.

17. Arber N, Spicak J, Rácz I, Zavoral M, Breazna A, Gerletti P, et al. Five-year analysis of the prevention of colorectal sporadic adenomatous polyps trial. Am. J. Gastroenterol. 2011;106:1135–46.

18. Bertagnolli MM, Eagle CJ, Zauber AG, Redston M, Breazna A, Kim K, et al. Five-year efficacy and safety analysis of the Adenoma Prevention with Celecoxib Trial. Cancer Prev. Res. Phila. Pa. 2009;2:310–21.

19. Takayama T, Nagashima H, Maeda M, Nojiri S, Hirayama M, Nakano Y, et al. Randomized double-blind trial of sulindac and etodolac to eradicate aberrant crypt foci and to prevent sporadic colorectal polyps. Clin. Cancer Res. Off. J. Am. Assoc. Cancer Res. 2011;17:3803–11.

20. Cooper K, Squires H, Carroll C, Papaioannou D, Booth A, Logan RF, et al. Chemoprevention of colorectal cancer: systematic review and economic evaluation. Health Technol. Assess. Winch. Engl. 2010;14:1–206.

21. Cole BF, Logan RF, Halabi S, Benamouzig R, Sandler RS, Grainge MJ, et al. Aspirin for the chemoprevention of colorectal adenomas: meta-analysis of the randomized trials. J. Natl. Cancer Inst. 2009;101:256–66.

22. Gao F, Liao C, Liu L, Tan A, Cao Y, Mo Z. The effect of aspirin in the recurrence of colorectal adenomas: a meta-analysis of randomized controlled trials. Colorectal Dis. Off. J. Assoc. Coloproctology G. B. Irel. 2009;11:893–901.

23. Wang Y, Zhang F-C, Wang Y-J. The efficacy and safety of non-steroidal anti-inflammatory drugs in preventing the recurrence of colorectal adenoma: a meta-analysis and systematic review of randomized trials. Colorectal Dis. Off. J. Assoc. Coloproctology G. B. Irel. 2015;17:188–96.

24. Zhao T-Y, Tu J, Wang Y, Cheng D-W, Gao X-K, Luo H, et al. The Efficacy of Aspirin in Preventing the Recurrence of Colorectal Adenoma: a Renewed Meta-Analysis of Randomized Trials. Asian Pac. J. Cancer Prev. APJCP. 2016;17:2711–7.
